# A Review of Gaucher Disease Pathophysiology, Clinical Presentation and Treatments

**DOI:** 10.3390/ijms18020441

**Published:** 2017-02-17

**Authors:** Jérôme Stirnemann, Nadia Belmatoug, Fabrice Camou, Christine Serratrice, Roseline Froissart, Catherine Caillaud, Thierry Levade, Leonardo Astudillo, Jacques Serratrice, Anaïs Brassier, Christian Rose, Thierry Billette de Villemeur, Marc G. Berger

**Affiliations:** 1 Department of Internal Medicine, Geneva University Hospital, Rue Gabrielle-Perret-Gentil 4, CH-1211 Genève, Switzerland; christine.serratrice@hcuge.ch (C.S.); jacques.serratrice@hcuge.ch (J.S.); 2 Department of Internal Medicine, Reference Center for Lysosomal Storage Diseases, Hôpitaux Universitaires Paris Nord Val de Seine, site Beaujon, Assistance Publique-Hôpitaux de Paris, 100 boulevard du Général Leclerc, F-92110 Clichy la Garenne, France; nadia.belmatoug@aphp.fr; 3 Réanimation Médicale, Hôpital Saint André, CHU de Bordeaux, 1 rue Jean Burguet, F-33075 Bordeaux, France; fabrice.camou@chu-bordeaux.fr; 4 Service de Biochimie et Biologie Moléculaire Grand Est, unité des Maladies Héréditaires du Métabolisme et Dépistage Néonatal, Centre de Biologie et de Pathologie Est, Hospices Civils de Lyon, F-69677 Bron, France; roseline.froissart@chu-lyon.fr; 5 Inserm U1151, Institut Necker Enfants Malades, Université Paris Descartes, Laboratoire de Biochimie, Métabolomique et Protéomique, Hôpital Universitaire Necker Enfants Malades, Assistance Publique-Hôpitaux de Paris, 149 rue de Sèvres, F-75005 Paris, France; catherine.caillaud@inserm.fr; 6 Institut National de la Santé et de la Recherche Médicale (INSERM) UMR1037, Centre de Recherches en Cancérologie de Toulouse (CRCT), Université Paul Sabatier, Laboratoire de Biochimie Métabolique, Institut Fédératif de Biologie, CHU Purpan, F-31059 Toulouse, France; levade.t@chu-toulouse.fr; 7 Institut National de la Santé et de la Recherche Médicale (INSERM) UMR1037, Equipe Labellisée Ligue Contre le Cancer 2013, Centre de Recherches en Cancerologie de Toulouse (CRCT), Université de Toulouse, Service de Médecine Interne, CHU Purpan, F-31059 Toulouse, France; leonardo.astudillo31@gmail.com; 8 Centre de Référence des Maladies Héréditaires du Métabolisme de l’Enfant et de l’Adulte (MaMEA), Hôpital Necker-Enfants Malades, Assistance Publique-Hôpitaux de Paris, Université Paris Descartes, Institut Imagine, F-75012 Paris, France; anais.brassier@nck.aphp.fr; 9 Service d’onco-hématologie, Saint-Vincent de Paul Hospital, Boulevard de Belfort, Université Catholique de Lille, Univ. Nord de France, F-59000 Lille, France; Rose.Christian@ghicl.net; 10 Service de Neuropédiatrie, Pathologie du développement, Sorbonne Université, Reference Center for Lysosomal Diseases, Hôpital Trousseau, Assistance Publique-Hôpitaux de Paris, 24 Avenue du docteur Arnold Netter, F-75012 Paris, France; thierry.billette@aphp.fr; 11 CHU Estaing et Université Clermont Auvergne, Hematology (Biology) et EA 7453 CHELTER, F-63000 Clermont-Ferrand, France; mberger@chu-clermontferrand.fr

**Keywords:** Gaucher disease, lysosomal storage disease, glucocerebrosidase, *GBA1* gene, enzyme replacement therapy, substrate reduction therapy, biomarkers

## Abstract

Gaucher disease (GD, ORPHA355) is a rare, autosomal recessive genetic disorder. It is caused by a deficiency of the lysosomal enzyme, glucocerebrosidase, which leads to an accumulation of its substrate, glucosylceramide, in macrophages. In the general population, its incidence is approximately 1/40,000 to 1/60,000 births, rising to 1/800 in Ashkenazi Jews. The main cause of the cytopenia, splenomegaly, hepatomegaly, and bone lesions associated with the disease is considered to be the infiltration of the bone marrow, spleen, and liver by Gaucher cells. Type-1 Gaucher disease, which affects the majority of patients (90% in Europe and USA, but less in other regions), is characterized by effects on the viscera, whereas types 2 and 3 are also associated with neurological impairment, either severe in type 2 or variable in type 3. A diagnosis of GD can be confirmed by demonstrating the deficiency of acid glucocerebrosidase activity in leukocytes. Mutations in the *GBA1* gene should be identified as they may be of prognostic value in some cases. Patients with type-1 GD—but also carriers of *GBA1* mutation—have been found to be predisposed to developing Parkinson’s disease, and the risk of neoplasia associated with the disease is still subject to discussion. Disease-specific treatment consists of intravenous enzyme replacement therapy (ERT) using one of the currently available molecules (imiglucerase, velaglucerase, or taliglucerase). Orally administered inhibitors of glucosylceramide biosynthesis can also be used (miglustat or eliglustat).

## 1. Introduction

Lysosomal storage diseases (LSDs) are a group of heterogeneous inherited diseases caused by mutations affecting genes that encode either the function of the lysosomal enzymes required for the degradation of a wide range of complex macromolecules, but sometimes the function of specific transporters needed to export degraded molecules from the lysosomes. The resulting lysosomal dysfunction leads to cellular dysfunction and clinical abnormalities. In one group of LSDs, the sphingolipidoses, there is a dysfunction in the enzyme-degrading abilities of the metabolites which are essential components of cell membranes and regulators of various signaling pathways [1].

## 2. Definition of Gaucher Disease

**Figure 1:**
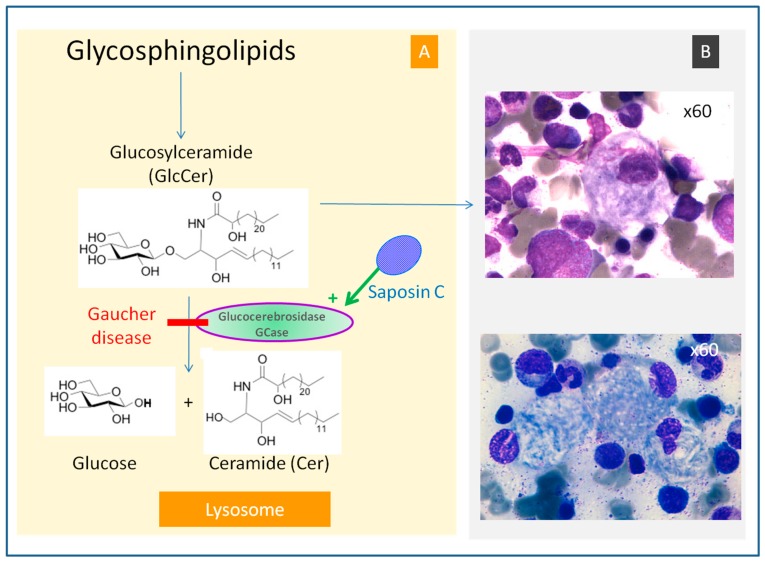
Hydrolysis of glucosylceramide (GlcCer) by glucocerebrosidase (GCase) in the lysosome (**A**). GCase is activated by saposin C. In lysosomal storage diseases, an enzyme deficiency is responsible for the accumulation of its substrate in the cell lysosome (overload disease). Gaucher disease is caused by a deficiency in glucocerebrosidase (GCase) (or β-glucosidase), which leads to an accumulation of GlcCer. GlcCer forms fibrillar aggregates that accumulate in macrophages and result in the cell cytoplasm presenting a characteristic “crumpled tissue paper” appearance (**B**), personal pictures, with the courtesy of Fabrice Camou and Rachid Seddik). These cells, known as Gaucher cells, infiltrate various organs (e.g., bone marrow, spleen, and liver) and are responsible for the major signs of the disease.

Gaucher disease (GD, OMIM #230800, ORPHA355) is the most common sphingolipidosis. It was first described by Philippe Gaucher in 1882 in a patient with massive splenomegaly without leukemia. GD is a rare, autosomal, recessive genetic disease caused by mutations in the *GBA1* gene, located on chromosome 1 (1q21). This leads to a markedly decreased activity of the lysosomal enzyme, glucocerebrosidase (GCase, also called glucosylceramidase or acid β-glucosidase, EC: 4.2.1.25), which hydrolyzes glucosylceramide (GlcCer) into ceramide and glucose (Figure 1A). More than 300 *GBA* mutations have been described in the *GBA1* gene [2]. Very rarely, GD can also be caused by a deficiency in the GCase activator, saposin C [3]. The phenotype is variable, but three clinical forms have been identified: type 1 is the most common and typically causes no neurological damage, whereas types 2 and 3 are characterized by neurological impairment. However, these distinctions are not absolute, and it is increasingly recognized that neuropathic GD represents a phenotypic continuum, ranging from extrapyramidal syndrome in type 1, at the mild end, to hydrops fetalis at the severe end of type 2 [4].

## 3. Epidemiology

The disease’s incidence is around 1/40,000 to 1/60,000 births in the general population, but it can reach 1/800 births in the Ashkenazi Jewish population [5,6].

## 4. Pathophysiology

### 4.1. Glucosylceramide Accumulation

Mutations in the *GBA1* gene lead to a marked decrease in GCase activity. The consequences of this deficiency are generally attributed to the accumulation of the GCase substrate, GlcCer, in macrophages, inducing their transformation into Gaucher cells. Under light microscopy, Gaucher cells are typically enlarged, with eccentric nuclei and condensed chromatin and cytoplasm with a heterogeneous “crumpled tissue paper” appearance (Figure 1B). This feature is related to the presence of GlcCer aggregates in characteristic twisted, fibrillar arrangements that can be visualized using electron microscopy [7]. Gaucher cells mainly infiltrate bone marrow, the spleen, and liver, but they also infiltrate other organs and are considered the main protagonists factors in the disease’s symptoms. The monocyte/macrophage lineage is preferentially altered because of their role in eliminating erythroid and leukocytes, which contain large amounts of glycosphingolipids, a source of GlcCer. GlcCer accumulation in Gaucher cells is considered the first step towards bone involvement, leading to the vascular compression which is the source of necrotic complications [8]. The pathophysiological mechanisms of neurological involvement remain poorly explained; GlcCer turnover in neurons is low and its accumulation is only significant when residual GCase activity is drastically decreased, i.e., only with some types of *GBA1* mutations [9]. Consistent with this, recent work on a Drosophila model of neuronopathic GD demonstrated autophagy impairment in the GCase-deficient fly brains [10]. Very rarely, GD may be caused by a mutation in the *PSAP* gene, leading to a deficiency in saposin C without GCase deficiency [3]. These patients generally present with neurological features similar to that of type-3 GD.

### 4.2. Subpopulation of Gaucher Cells, a Specific Cell Subpopulation

Recent observations indicate that Gaucher cells do not only result from the transformation of macrophage cells, but correspond to a distinct M2 subpopulation from an alternative differentiation pathway [11]. There are many functional states of macrophage polarization, and they can be fully polarized and acquire a specific phenotype like M1 (characteristic macrophage activation) or M2 (alternative macrophage activation). These specific phenotypes depend on the tissue and specific microenvironment where the macrophages are. The M2 subpopulation has been described as cells with anti-inflammatory, immunomodulatory and tissue repair properties, and includes macrophages that remove abnormal hematopoietic cells or phagocytose erythroblast nuclei. The in vivo situation appears more complex since the plasma cytokine profile and the characteristic monocytes circulating in the blood show concurrent activation of inflammatory M1 macrophages, presumably implicated in the “pseudo-inflammatory” state that was described many years ago and in the heterogeneous manifestations of the disease [12,13]. Thus numerous cytokines, chemokines and other molecules—including IL-1β, IL-6, IL-8, TNFα (Tumor Necrosis Factor), M-CSF (Macrophage-Colony Stimulating Factor), MIP-1β, IL-18, IL-10, TGFβ, CCL-18, chitotriosidase, CD14s, and CD163s—are present in increased amounts in Gaucher patients’ plasma and could be implicated in hematological and bone complications [14,15,16,17]. Only some of these molecules are expressed by Gaucher cells themselves. This is the case for chitotriosidase and CCL18, which thus constitute quite specific disease biomarkers [11]. Osteoporosis may be linked to IL-10, which inhibits osteoblast activity, but also to IL-1β, IL-6 and M-CSF, MIP-1α and MIP-1β, which stimulate bone resorption by increasing osteoclast activity [14,17].

The relationship between Gaucher cells located in tissues and developed from M2 macrophages and blood monocytes with an M1 phenotype is still not understood, and this distinction should be maintained.

### 4.3. Metabolic Consequences Other Than Accumulation of Glucosylceramide in Gaucher Cells

**Figure 2:**
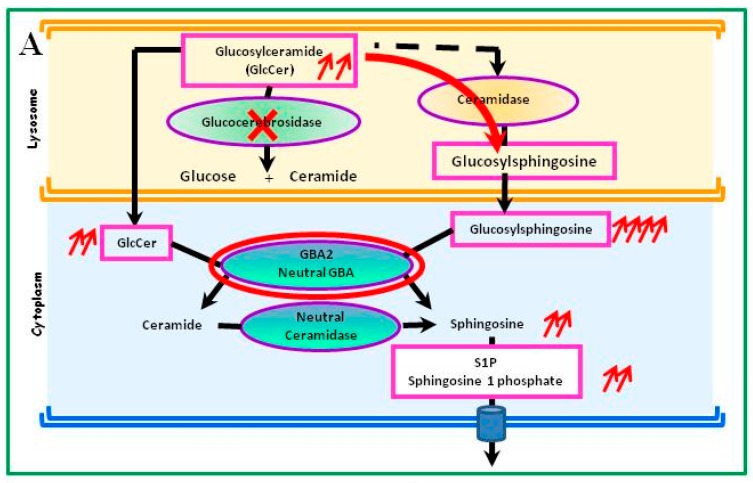

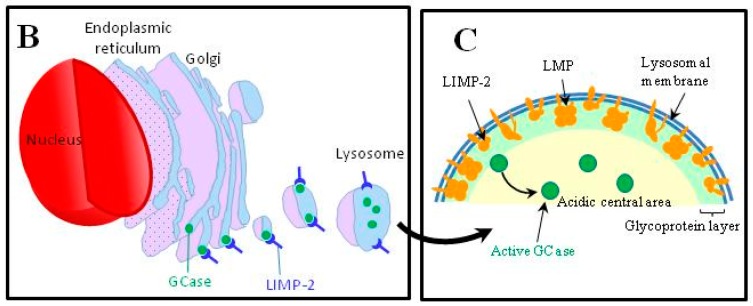
Alternative metabolic pathway of the glucosylceramide (GlcCer) accumulation due to the glucocerebrosidase (GCase) deficiency. The expression of GCase varies from one cell type to another and depends on the tissue. (**A**) In a mouse model of GCase deficiency (red cross), GlcCer is transformed via an alternative ceramidase pathway into glucosylsphingosine (red arrow), which is degraded by cytoplasmic GCase2 (*GBA2* gene), active at a neutral pH, to S1P, a very active metabolite [20]. (**B**) Protein maturation takes place in the Golgi apparatus; the transport and delivery of GCase to lysosomes require a particular molecule, LIMP-2, which allows GCase to reach the lysosome where the acidic pH breaks the molecular link [39]. (**C**) LIMP-2 is a lysosomal membrane protein (LMP) whose highly glycosylated intra-lysosomal part protects the lysosome’s membrane. LIMP-2 anomalies can induce a phenotype rather than GD3 [39].

Due to the accumulation of GlcCer, Mistry et al. identified another metabolic pathway in a mouse model [18] (Figure 2A). GlcCer is also the substrate of an alternative pathway in which a ceramidase transforms it into glucosylsphingosine (or Lyso-glucosylceramide), which then diffuses into fluids due to its reduced hydrophobicity. This pathway is favored in cases of GCase deficiency. In the cytoplasm, glucosylsphingosine is metabolized by a second GCase that is active at a neutral pH (*GBA2* gene), producing sphingosine and then sphingosine-1-phosphate (S1P) [19,20]. Sphingosine could be particularly toxic to bone; in this model, deletion of *GBA2* could reverse the Gaucher disease phenotype, particularly the bone abnormalities. In addition, the accumulation of glucosylsphingosine may cause neuronal dysfunction and death, leading mainly to GD-related neurological symptoms [21]. Glucosylsphingosine is normally absent from the human brain, but it is detectable in the brains of patients with GD-related neurological lesions, even if Gaucher cells are not observed in their nervous system. Glucosylsphingosine could represent a more specific and sensitive biomarker than chitotriosidase or CCL18 [19,22]. Glucosylsphingosine could serve as a source of S1P, influencing the differentiation, migration, and survival of several cell types, including lymphocytes and macrophages [23].

It has been demonstrated that the enzyme deficiency may have an impact on many cells, including hematopoietic progenitor cells, erythrocytes or mesenchymal cells [24,25,26], hepatocytes [27] and the nerve cells of patients with neurological lesions.

### 4.4. Abnormalities in the Intracellular Trafficking of Glucocerebrosidase

The enzymatic deficit of GCase is not only due to the intrinsic enzymatic dysfunction but is also the consequence of abnormalities occurring during the transport and delivery of the enzyme to the lysosomes. Thus, enzyme misfolding during its passage through the endoplasmic reticulum can lead to its premature degradation by the proteasome [28,29] (Figure 2B–C).

The transport and delivery of GCase to the lysosome does not depend on the mannose 6-phosphate system, like other proteins, but also involves Lysosomal Integral Membrane Protein 2 (LIMP-2) [30]. GCase linked with LIMP-2 is inactive. The acidic milieu of lysosomes promotes this delinking, allowing its activation. *LIMP-2* mutations (*SCARB2* gene) have been described in several neurological disorders [31,32]. LIMP-2 mutations can affect the GD phenotype [33]. It seems that while LIMP-2 deficiency alone does not cause the observed phenotype, it is probably an important modifier of GD, potentially turning a patient with type-1 GD into a type-3 [33]. SCARB2 mutations could explain GD heterogeneity in the context of the same mutation of GCase. Other molecules could be involved in this trafficking pathway of GCase, such as progranulin. Progranulin is an indispensable co-chaperone that links GCase/LIMP2. The serum level of progranulin is significantly lower in GD patients than in healthy controls, and this leads to GCase accumulation in the cytoplasm [34]. The loss of progranulin leads to abnormal endothelial reticulum trafficking and the aggregation of various proteins in the cytoplasm, such as GCase/LIMP2, which increase degradation of GCase [35]. An insufficiency of PGRN has also been associated with many types of neurodegenerative disease, including frontotemporal dementia, Parkinson’s disease (PD), Alzheimer's disease, multiple sclerosis, and amyotrophic lateral sclerosis [36].

It has been observed that patients with the same GCase mutations may vary significantly in disease presentation, from life-threatening to almost asymptomatic [37,38], because of molecular co-abnormalities. This helps us to understand the phenotypic heterogeneity of GD.

### 4.5. Relationship between the GBA1 Gene and Parkinson’s Disease

**Figure 3:**
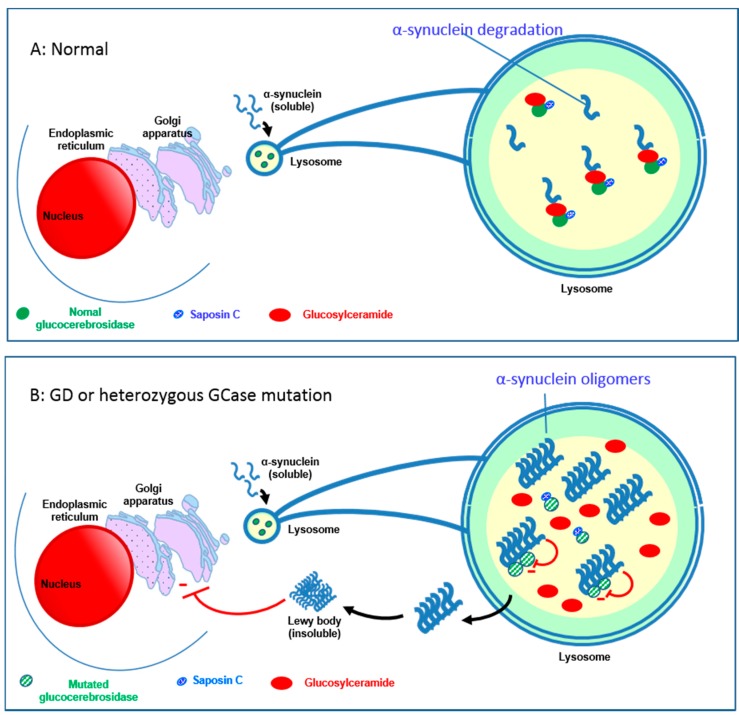
Relationship between glucocerebrosidase (GCase) and neurological diseases with Lewy bodies. (**A**) Normally, GCase interacts with its substrate glucosylceramide (GlcCer) as well as monomers of α-synuclein in lysosomes, facilitating the breakdown of both at acidic pH; (**B**) Mutated GCase or decreased levels of GCase result in a slowdown of α-synuclein degradation and a gradual build up of GlcCer, with the formation of α-synuclein oligomers and fibrils [48,49,54]; GlcCer stabilizes the α-synuclein oligomers [47]. These oligomers are able to bind to the mutated GCase molecules and inhibit the enzymatic activity of GCase, further decreasing the enzyme activity [47,50,55]. These impaired lysosomes show impaired chaperone-mediated autophagy and autophagosome fusion. This results in an increased accumulation of α-synuclein in the cytoplasm, forming insoluble aggregates to form Lewy bodies. These aggregates block trafficking of GCase from the endoplasmic reticulum (ER) to the Golgi [56]. Mutant GCase is retained in the Endoplasmic reticulum, which causes ER stress and evokes the ER stress response (Unfolded Protein Response) [57]. Saposin C can have a modulating effect on this by binding to GCase and thus maintaining its activity [51,58].

Patients with a heterozygous (or homozygous) mutation in the *GBA1* gene, especially c.1226A>G (N370S), but also c.1448T>C (L444P), c.84dup, c.115+1G>A (IVS2+1G>A), c.1297G>T (V394L), and c.1604G>A (R496H), are now considered at risk for Parkinson’s disease (PD) [40,41,42]. However, all GBA mutations including null alleles seem to increase the risk for PD [43]. The onset of PD tends to be earlier in patients carrying null or recombinant alleles [44]. The prevalence of heterozygous mutations is found to range from 3% to 8% in Caucasian PD [42,43] and is higher in the Ashkenazi Jewish population, reaching 15% or even 31% [40,43]. The most recent studies suggest that neuropathic mutations of the *GBA* gene (especially c.1448T>C (L444P)) could worsen the progression of PD [45,46]. Loss of GCase function compromises lysosomal α-synuclein degradation and causes accumulation of oligomers. It results in neurotoxicity through accumulation in the substantia nigra of the brain. GlcCer, the GCase substrate, directly influences amyloid formation of α-synuclein by stabilizing soluble oligomers, which then aggregate and form Lewy bodies in the nerve cells in PD [47]. These α-synuclein polymers have an inhibitory effect on GCase [47,48,49], engendering a vicious circle (Figure 3). The effect of *GBA1* gene mutations could be modulated by the co-activator of GCase, saposin C, which may partly explain the limited penetrance of neurological impairment in patients with GD [50,51]. GCase deficiency has been implicated in non-*GBA1* linked PD, and interaction between GCase and other molecules involved in PD pathophysiology has been described [52,53]. GCase levels decrease with age in non-PD patients and GCase is decreased in idiopathic PD as well as *GBA1*-linked PD.

### 4.6. Relationship between GCase Deficiency and Neoplasia

The frequency of hypergammaglobulinemia and the presence of monoclonal Ig in GD are two factors which promote the emergence of multiple myeloma; the incidence of myeloma appears to be increased in GD, with a relative risk of at least 5.9 (95% CI: 2.8–10.8) [59,60,61,62].

There is also an increased relative risk of lymphoma [61,63] and of solid cancer (hepatocellular carcinoma [62], melanoma, and pancreatic cancer [63]), but there is less evidence than for hematological cancers.

The pathophysiology of cancer development in GD is not well understood. At least two types of mechanisms may be operating, both of them relating to the GCase deficiency and the ensuing catabolic defect, i.e., the accumulation of GlcCer and/or its deacylated product, glucosylsphingosine or Lyso-glucosylceramide (LGL1) [64].

The most common hypothesis is that the (perturbed) cellular and cytokinic microenvironment in GD is responsible for tumorigenesis: perturbations include markedly elevated levels of some cytokines and chemokines [14,15,16,17], activated (M2) macrophages [11], abnormal responses by T lymphocytes, and a reduction of NK cells [65,66]. The second hypothesis considers that initial steps towards a catabolic defect originate not from the environment, but from the (future) malignant cell itself. Facilitation of tumorigenesis in GD could be related to the disturbed sphingolipid metabolism in cancer cells, due to GlcCer (or glucosylsphingosine) accumulation or reduced ceramide formation, resulting in deleterious changes in the pro- and anti-proliferative balance [64].

Recently, a murine model of GD with a long-term development of B cell malignancies has been investigated [67]. Interestingly, mice bearing large tumors had less GlcCer and glucosylsphingosine accumulation than those with small tumors. In this model, the monoclonal protein disappeared from mice treated with eliglustat; moreover, a striking reduction in lymphoproliferation was observed, with no plasmocytoma or lymphoma [68]. A more recent study on monoclonal immunoglobulin (Ig) in GD showed that in 17 of 20 patients with GD and six of six Gaucher mice, the clonal Ig was specific for glucosylsphingosine. Given that myeloma plasma cells showed evidence of antigen-driven selection and that there is increased risk of MGUS and myeloma in GD, it was tested whether immunoglobulin was reactive to LGL1 and lysophosphatidylcholine. In 33% of sporadic monoclonal gammopathies, the clonal Ig was specific for the two targets. Thus, extended exposure of the immune system to high levels of LGL1 and lysophosphatidylcholine might favor gammapathies and myeloma [69]. This observation suggests that LGL1 could be a relevant predictive biomarker and should be further studied in this context. Moreover, treating the above murine model of GD with eliglustat led to a reduction of clonal Ig. The glucosylsphingosine might mediate B-cell activation in GD and might indeed be the antigenic origin of GD-associated monoclonal gammopathy. These new data give glucosylsphingosine a key role in myeloma or lymphoma in GD [69].

### 4.7. Altered Iron Metabolism

Iron is stored in ferritin to avoid toxicity to cell components. In GD, ferritin levels in Gaucher cells are higher and the synthesis of hepcidin, which inhibits intestinal absorption of iron, is increased. In Gaucher cells, certain cytokines (IL-6 and IL-1β) also increase hepcidin gene transcription; the macrophages activated in this way can also induce iron retention via an autocrine mechanism [70] and decrease glycosylation capabilities, leading to a decrease in glycosylated ferritin [71]. Hyperferritinemia in type-1 GD is associated with indicators of disease severity [72], and ferritin could be a useful though nonspecific biomarker; it is also an inflammation marker.

## 5. Clinical Presentations

Gaucher disease is characterized by hepatosplenomegaly, cytopenia, sometimes severe bone involvement and, in certain forms, neurological impairment. The variability in the clinical presentations of GD may be explained by the continuum of phenotypes [73]. However, three major phenotypic presentations can usually be distinguished. They are described below, in order of increasing severity. Type-1 GD is usually named non-neuronopathic GD; type-2 and type-3 are termed neuronopathic-GD.

### 5.1. Type-1 Gaucher Disease (ORPHA77259)

Type-1 GD (GD1), usually distinguished by the absence of neurological impairment, is the most common form of the disease (prevalence: 90%–95% in Europe and North America). Its clinical presentation is variable, ranging from asymptomatic throughout life to early-onset forms presenting in childhood. The initial symptoms vary considerably and patients can be diagnosed at any age [6]. Depending on the study, the median age of diagnosis is from 10 to 20 years old [6,74]. Although the overall mean onset of patients in the Gaucher Registry (run by the International Collaborative Gaucher Group) is at 20.4 years old, the majority (56%) of patients experienced onset before 20. However, this Registry primarily includes symptomatic and treated patients, and thus the mean age is probably skewed. Two thirds (68%) of this group were diagnosed before 10 years old and almost half (48%) before the age of 6 [75,76]. GD1 can often limit quality of life and is often associated with considerable morbidity, but is rarely life threatening.

Fatigue is common (50% of patients) and often has an impact on school life or socio-professional activities. In children, growth retardation and delayed puberty are common (growth <5th percentile in 34% of children) [75].

Splenomegaly is observed in more than 90% of patients and is sometimes massive, with a spleen weighing up to several kilograms and causing abdominal pain or distension. Indeed, it may be the only clinical sign, leading to unnecessary tests if GD is not considered. Splenic infarction may complicate matters; spleen rupture only occurs very rarely [77,78].

Hepatomegaly is noted in 60%–80% of patients. The development of fibrosis and subsequent cirrhosis is rare [6]. Hepatic and splenic infarction may be observed, manifesting with acute pseudo-surgical abdominal pain.

Up to 40% of GD1 patients have a focal lesion in the liver and/or spleen. A *gaucheroma* is the most likely diagnosis, but a hepatocellular carcinoma or a lymphoma of the spleen are other possible diagnoses. Gaucheromas have varied imaging characteristics and it is therefore difficult to distinguish a gaucheroma from another lesion [79].

The prevalence of *gallstones* in GD1 is 32%, i.e., five times higher than in the general population [27]. Bile analyses reveal cholesterol stones and GlcCer.

Bleeding phenomena may be observed at diagnosis. These are rarely severe and usually related to thrombocytopenia (60%–90% of cases) or to coagulation or primary hemostasis disorders [80] or, more rarely, to platelet disorders [81]. Mucocutaneous bleeding (epistaxis, gingival bleeding, menorrhagia, etc.) is common; postoperative hemorrhage or bleeding during birth and spontaneous hematomas (e.g., psoas hematomas) have also been reported. Anemia, observed in 20%–50% of cases, is generally moderate. Leukopenia is rare.

Bone involvement causes acute pain manifested as very painful bone crises, predominantly in the pelvis and lower limbs (more rarely in the upper limbs), and/or chronic pain that should be assessed using a visual analog scale or digital scale [82]. The severity of the pain varies, but it may have an impact on functional prognosis. The pathophysiology of bone manifestations is poorly known and justifies the use of common terminology. The painful bone crises are probably associated with ischemic vaso-occlusive phenomena. It seems that they may be reversible and do not show up as lesions in medical imaging. However, they usually cause abnormalities referred to as bone infarcts on long bones (metaphyses or diaphyses) and flat bones, and lesions referred to as avascular necrosis (AVN) on the epiphyses. In addition to the vascular theory explaining the ischemic events (bone infarcts and AVN), a mechanical theory has also been put forward to explain the spontaneous or trabecular microfractures that are observed (mechanical or spontaneous AVN), particularly on the femoral head, the femoral condyle and the tibial plateau. The pathophysiological mechanism can involve other potential factors, such as alterations in the bone marrow or immune cells, inflammation, macrophage-derived factors, cytokines, and hormones [83,84].

Acute painful bone crises are more common in children (30% of children with GD1). They usually progress over 7–10 days and are associated with local inflammation, mild fever (38 °C), polynuclear leukocytosis and a moderate inflammatory syndrome. These symptoms are similar to osteomyelitis (pseudo-osteomyelitis), thus sometimes delaying diagnosis [82,85]. AVN is observed in 15% of cases, most often at the femoral or humeral heads, more rarely at the femoral condyles or tibial plateaus, and exceptionally in the feet (talus, calcaneus), hands and the spine (vertebra plana). In the long term, AVN may be complicated by osteoarthritis, often justifying joint replacement surgery [72]. Bone crises are predictive of future bone infarcts and AVN. Bone infarcts with clinical consequences, AVN and pathological fractures are considered as severe bone complications of GD and are defined as bone events [72].

Moderate losses of bone mass (osteopenia) or more severe declines (osteoporosis) increase with age and menopause in normal subjects. Loss of bone mass occurs earlier and is more severe in patients with GD and may cause pathological fractures (of long bones, vertebrae, etc.). Bone mass decline seems to be correlated with other bone and visceral complications [86]. Focal lytic lesions that can erode the cortical bone and promote pathological fractures are sometimes observed in different locations (long bones, jaw, etc.) [87]. Cyst-like lesions in the mandible, with the loss of trabecular structure, may lead to dental abnormalities. Extra-osseous extension of Gaucher cells secondary to the destruction of the cortical bone only occurs very rarely [88]. Secondary bone tumors including osteosarcomas or osteoblastomas have been reported very rarely [89,90].

When magnetic resonance imaging (MRI) is not available, standard radiographs may be used to observe bone remodeling disorders with enlargement of the metaphyseal/diaphyseal region of the femur’s lower part, referred to as the Erlenmeyer flask bone deformity, appearing during childhood [91]. The sequelae of AVN and bone infarction, thinning of the cortical bone, focal lysis, fractures and osteoarthritis can also be observed. Radiographs may also be used to monitor patients after joint replacement surgery. MRI is the reference examination and is used to objectify bone marrow infiltration (80% of cases) at a very early stage, as well as bone infarcts, AVN and bone lysis. Bone marrow infiltration and Erlenmeyer flask bone deformities do not seem to correlate with the other bone complications [92].

Pulmonary involvement may be related to infiltration of the lungs by Gaucher cells, creating an interstitial disease that can lead to pulmonary fibrosis, restrictive lung disease secondary to spinal deformation, or pulmonary arterial hypertension [93,94]. The latter is more common in splenectomized patients, particularly women, or may be caused by hepatopulmonary syndrome complicating hepatic cirrhosis. Pulmonary involvement is rare in all GD phenotypes and seems more frequent in patients homozygous for the 1448G (L444P) mutation [95].

Rarely, renal involvement, manifested as proteinuria and hematuria, reflects infiltration of glomeruli by Gaucher cells [96]. Skin involvement is manifested as yellow-brown hyperpigmentation, usually on the anterior parts of the tibias and cheeks [97]. Ocular manifestations such as vasculitis [98,99] or vitreo-retinal involvement with whitish spots (corresponding to glucoceramide deposits) [98,100], myocardial or valvular involvement [101], insulin resistance [102] and amyloidosis [103,104,105] are observed very rarely.

Contrary to the conventional definition of GD1, certain neurological manifestations associated with this phenotype have been described in recent years. Patients with GD1 have an increased risk of developing Parkinson’s disease (4–20 times greater), often at an earlier age than in normal PD [43,47,106,107]. The prevalence of minimally symptomatic peripheral neuropathies and small fiber neuropathies is 14% and therefore higher than in the general population [108].

### 5.2. Type-3 Gaucher Disease (ORPHA77261)

Also called juvenile or subacute neurological GD, the type-3 form (usually 5% of cases, but up to 33% in some cohorts [109]) exhibits the visceral manifestations described in GD1, usually combined with oculomotor neurological involvement, which appears before 20 years of age in most cases. Like GD1, GD3 phenotypes are very heterogeneous, particularly with regard to neurological involvement. Some patients present moderate systemic involvement with horizontal ophthalmoplegia as the only neurological symptom, whereas others present more severe forms with varying neurological signs including progressive myoclonus epilepsy (16% of patients), cerebellar ataxia or spasticity (20%–50% of patients), and dementia in some cases [110,111]. Neurological signs may occur several years after the visceral manifestations, even in patients initially identified and treated as having GD1. Disease onset is more common in young children, with neurological symptoms appearing before 2 years of age in half the cases [110]. Behavioral changes and unexpected death are described in some patients [112].

Spinal surgery may be required for the sometimes severe and progressive kyphosis that may develop, through an unknown mechanism, despite specific GD treatment. Cardiac involvement (with valve calcification) [113], corneal involvement and hydrocephaly are reported mainly in patients with GD3 of the c.1342G>C (D409H) genotype [114].

Subjects with the very rare saposin C deficiency almost always present with neurological impairment comparable to that observed in GD3 [115].

### 5.3. Type-2 Gaucher Disease (ORPHA77260)

Type-2 GD (<5% of cases in most countries, but up to 20% in some cohorts [109]) is characterized by early and severe neurological impairment starting in infants aged 3–6 months old and by systemic involvement with hepatosplenomegaly. The triad consisting of rigidity of the neck and trunk (opisthotonus), bulbar signs (particularly severe swallowing disorders), and oculomotor paralysis (or bilateral fixed strabismus) is very suggestive of the disease. These signs may be associated with trismus and hypertonia with pyramidal and possibly extrapyramidal rigidity [116].

Apnea related to increasingly frequent and lengthy laryngeal spasms occurs after a few months. Psychomotor development is then altered, although some children may still continue to acquire skills. Seizures occurring later manifest as myoclonic epilepsy that is resistant to antiepileptic drugs. Splenomegaly is almost always present, associated with thrombocytopenia in 60% of cases. Growth retardation (30% of patients) may be the first sign, sometimes associated with cachexia. Lung lesions are sometimes also observed, resulting from repeated aspirations and pulmonary infiltration by Gaucher cells. There is no bone involvement in GD2. Death occurs before the third year of life, following massive aspiration or prolonged apnea [116,117]. The mean survival age of GD2 is 11.7 months (range 2–25 months), and pulmonary symptoms (GD-pneumopathy) and aspiration caused by Gaucher disease or the aggravation of respiratory conditions such as central apnea are the cause of 50% of fatal cases [116].

Fetal GD is the rarest (<1%) and the most severe form of the disease. It usually manifests with hydrops fetalis, hepatosplenomegaly, ichthyosis, arthrogryposis, facial dysmorphia and fetal thrombocytopenia. Death often occurs in utero or soon after birth [118]. The diagnosis of these fetal forms is particularly important for appropriate genetic counseling and the possibility of offering a prenatal diagnosis in future pregnancies.

## 6. Diagnosis of Gaucher Disease

The diagnosis of GD often takes place several years after the onset of the first clinical and laboratory signs. This is a typical problem with rare diseases characterized by a progressive onset of symptoms.

### 6.1. GCase Activity

The diagnosis of GD must be confirmed by establishing deficient GCase activity in total leukocytes or mononuclear cells, or cultured fibroblasts. The residual enzyme activity is usually approximately 10%–15% of the normal value [119].

Dried blood spots can also be used for the enzymatic assay, but any potential deficiency should be confirmed using the previous method. Flow cytometry analysis of blood monocytes is a more accurate method [26,120], but it has not been validated by different centers. The very rare saposin C deficiency should be tested for when GCase activity is normal but the clinical picture and biomarkers point to GD and especially when chitotriosidase activity is very high. The diagnosis is made by *PSAP* gene sequencing.

### 6.2. Bone Marrow Aspiration

Bone marrow aspiration is not mandatory to confirm a diagnosis of GD, but it may be performed on patients without a diagnosis when isolated thrombocytopenia and/or splenomegaly are found and it can help when Gaucher cells are found. However, bone marrow aspiration should not routinely be performed in GD. Very rarely, it may be impossible to use cytology for diagnosis when only a few cells are available. In addition, it may be difficult to distinguish Gaucher cells from the so-called “pseudo-Gaucher” cells observed in some blood disorders or infectious diseases, such as myeloma with histiocytic accumulation of immunoglobulin crystals [121], Waldenstrom’s disease and other lymphomas with monoclonal gammopathy [122], chronic myeloid leukemia or myelodysplasia [123,124], or atypical mycobacteria [125].

### 6.3. GBA1 Mutations

The gene encoding GCase (*GBA1*) is located on the long arm of chromosome 1 (1q21) and it contains 11 exons. The presence of a highly homologous pseudogene (*GBAP*) at the same locus (16 kb downstream) is responsible for recombination events between *GBAP* and *GBA1* (e.g., RecNciI allele) [126]. More than 400 mutations have been described in the *GBA1* gene, but some of them are more common, such as c.1226A>G (N370S), c.1448T>C (L444P), c.84dup, c.115+1G>A (IVS2+1G>A) and RecNciI [127]. The mutations most prevalent (90% of the mutant alleles) in Ashkenazi Jewish GD1 patients are c.1226A>G, c.84dup, c.1448T>C and c.115+1G>A, whereas they account for about 60% of total mutations in non-Ashkenazi patients. The c.1226A>G (N370S) mutation is rarely seen in Asian and Arab populations and mutant allele frequencies are very different.

The c.1226A>G (N370S) mutation is particularly common in the Ashkenazi Jewish population (about 75%–80% of the alleles), but it accounts for only 30% of the alleles in non-Jewish patients. The presence of the c.1226A>G (N370S) mutation in a homozygous or heterozygous state excludes the risk of neurological involvement (GD2 or GD3), but it does not predict the severity of bone and visceral involvement. Patients homozygous for the N370S mutation can remain asymptomatic for a long time, whereas those homozygous for the L444P mutation are at a high risk of developing neurological impairment (GD2 or GD3). Homozygotes for the rare c.1342G>C (D409H) mutation present characteristic heart valve damage [114]. Patients carrying two null mutations (leading to a total absence of glucocerebrosidase activity) do not survive beyond the perinatal period (fetal forms incompatible with life) [76].

### 6.4. Prenatal Diagnosis

Prenatal diagnosis of GD can be performed by genetic analysis, using either chorionic villus sampling (sampled at 10–12 weeks of amenorrhea) or amniotic fluid cells (as early as 16 weeks of amenorrhea), but only if the index case genotype has been previously identified [128]. It can also be done by measuring glucocerebrosidase activity on fresh chorionic villi or cultured amniotic cells.

## 7. Laboratory Abnormalities

### 7.1. Hemogram

Thrombocytopenia of varying degrees is common (90% of cases): <60 × 10^9^ platelets/L in 26% of cases; <120 × 10^9^ platelets/L in 76% of cases [74]. Anemia is less common (56% of cases) and moderate, with hemoglobin levels rarely found to be less than 9 g/dL; leukopenia is rare. Cases of GD without thrombocytopenia are observed. These cytopenias are attributed to splenic sequestration and bone marrow infiltration, but a direct impact of the enzyme deficiency on immature hematopoietic cells has also been described [26,129]. The blood count may be normal in patients with a history of splenectomy. Immune thrombocytopenias have been described.

### 7.2. Hemostasis

Several hemostatic abnormalities have been described in GD, including prolonged prothrombin time (PT) and activated partial thromboplastin time (APPT). These could possibly be related to deficiencies in factor X, factor V, thrombin (or a more global deficiency), factor XI (common among Ashkenazi Jews) or vitamin K. They could also be related to deficiencies secondary to liver failure (rare in GD) or to potentially associated genetic or even acquired von Willebrand disease [130]. However, the relationship with potential signs of bleeding is not obvious, especially as platelet disorders are common [131].

### 7.3. Proteinemia, Serum Immunofixation and Electrophoresis

These examinations must be carried out to screen for polyclonal hypergammaglobulinemia (25%–91% of cases) and possible monoclonal gammopathy (1%–35% of cases) [132,133]. Specific treatment reduces polyclonal hypergammaglobulinemia, but seems to have a limited effect on monoclonal gammopathy [134].

### 7.4. Disease Biomarkers

Currently, the most interesting biomarkers are chitotriosidase, CCL18, glucosylsphingosine and ferritin. Chitotriosidase is produced in large quantities by Gaucher cells, and it has been used as a biomarker since 1994 [135]. Its level is generally very high without treatment, so it can be used to monitor treatment efficacy and is considered to have some prognostic value [136]. However, chitotriosidase levels can vary considerably among patients; indeed, a mutation (24-bp duplication) in the *CHIT1* gene leads to total deficiency (homozygosity for the mutation) in 6% of the general population and chitotriosidase activity is low and difficult to interpret in a third of patients with a heterozygous mutation. In practice, these limitations hamper its use for between-patient comparisons [137]. Furthermore, different techniques used for the assessment of chitotriosidase levels prevent the comparison of results between centers. Increased chitotriosidase levels are also observed, but to a lesser extent, in certain other lysosomal storage diseases (e.g., Niemann–Pick diseases) and a variety of non-LSD disorders (sarcoidosis [138], β-thalassemia, multiple sclerosis, Alzheimer’s disease, or visceral leishmaniasis [139]).

CCL18 is a chemokine produced by various cell types, particularly macrophages (mainly of the M2 type) and dendritic cells [140]. CCL18 promotes the recruitment of T lymphocytes through CCR8 [141]. Gaucher cells produce CCL18 and it is found at high levels in plasma. CCL18 levels may also be increased in chronic inflammatory diseases such as idiopathic pulmonary fibrosis, some cancers and scleroderma; it is generally a bad prognosis [142,143]. Its levels are also increased in allergic reactions, insulin resistance and obesity. In GD patients, CCL18 plasma levels are 10–50 times higher than those of controls [11,144,145]. Its levels vary less than those of chitotriosidase since there is no genetic polymorphism; the kinetics of CCL18 and chitotriosidase are generally similar at treatment induction. High levels of CCL18 are associated with a poorer prognosis. It is essential to evaluate its level in patients with chitotriosidase deficiency. A standardization of the CCL18 assay should also be done before comparing data from different centers.

Glucosylsphingosine is a novel biomarker whose sensitivity and specificity are superior to those of chitotriosidase and CCL18 [19,22]. It was recently shown to be very valuable for patient monitoring [146,147,148], but has yet to be assessed on a larger scale. Assays are recommended at the same frequency as the other biomarkers.

All three biomarkers—chitotriosidase, CCL18, and glucosylsphingosine—are closely related within the context of GD: they vary in the same direction and are generally correlated [148].

Ferritinemia is higher than normal in most GD patients (>85%), while serum iron, transferrin saturation and soluble transferrin receptor concentrations remain normal [149]. High levels of iron reserves accumulate preferentially in the liver and bone marrow. Ferritin levels may be predictive of the onset of bone complications and are frequently associated with splenectomy [72]. The ferritin level should therefore be monitored in GD (even if it is a non-specific marker), as should increased inflammation (by monitoring for normal levels of C-reactive protein). Phlebotomy is theoretically not indicated for hyperferritinemia in GD; patients can be tested for associated hemochromatosis if transferrin saturation is high [150].

More accurate techniques, such as flow cytometry, can be used to quantify GCase enzyme activity in specific cell populations. This method is more sensitive because it is possible to focus the GCase assay on monocytes, where the level of enzyme is markedly higher than in other cells. This can reveal activities of the order of only 10% of normal activity [26,120]. The enzyme level could be used as a biomarker for treatment management.

The oldest biomarkers of GD are tartrate-resistant acid phosphatase (TRAP) and angiotensin converting enzyme (ACE). Their lack of specificity and the availability of more specific biomarkers have rendered them less useful today [151].

### 7.5. Others Biological Tests

Liver function tests (e.g., free and conjugated bilirubin, transaminases, alkaline phosphatase, gamma GT) are not usually abnormal but may be carried out, sometimes revealing cholestasis (increase in alkaline phosphatase, bilirubin, and gamma-GT), but rarely cytolysis (increase in transaminases).

C Reactive Protein (CRP) levels may be high in bone crises (bone infarction) or infectious complications (cholecystitis more common in GD).

Measurement of serum calcium (potentially serum phosphorus) and vitamin D is recommended. Vitamin D deficiency seems to be more common in GD than in the general population and supplementation is highly recommended when the 25(OH)D level is less than 75 nmol/L [152].

Auto-antibodies (antinuclear, anti-phospholipid antibodies) have been found in GD patients, usually without clinical signs, but are not routinely tested for. Antibodies directed against the therapeutic enzyme (imiglucerase) are detected in 2%–14% of cases, but are of no consequence in practice. They are only assayed in the case of an allergic reaction or a loss of treatment efficacy [153].

Some bone remodeling markers can be assayed: in theory, a decrease should be observed in bone formation markers (e.g., osteocalcin), whereas bone resorption markers (such as ICTP for C*-*terminal type-I collagen telopeptide) should be normal or higher, but the published studies are discordant [84].

Compared to control subjects, GD1 patients showed decreased HDL-cholesterol and ApoA1 levels, with increased triglyceride levels, however, there was no difference in the mean carotid intima-media thickness between GD patients and control subjects (not leading to premature atherosclerosis) [154].

## 8. Radiological Investigations

Abdominal magnetic resonance imaging (MRI) is the most appropriate examination for assessing liver and spleen dimensions (organ volume) and morphology. The spleen sometimes presents nodules suggestive of lymphoma. When MRI is unavailable or in cases of uncontrollable claustrophobia, an abdominal ultrasound may be used instead. Computed tomography (CT) has previously been used for estimating Gaucher organ volumes; nonetheless, MRI, because of justifiable concerns about CT radiation, is preferable because repeat assessments are routinely required [155].

Bone magnetic resonance imaging (MRI) is the test of choice for evaluating the effects of GD on bone. Bone marrow infiltration is predominant at the proximal and distal ends. T1 weighted sequences are recommended to detect and quantify bone marrow infiltration, while T2 weighted sequences are used to detect complications such as AVN or bone infarction [156]. Hypointense signals are generally observed in T1 weighted sequences, reflecting the replacement of normal bone marrow fat by Gaucher cells. Infiltration may be quantified by means of the various scores used in reference centers, such as the Bone Marrow Burden score [157,158]. Assessment of bone marrow infiltration is more difficult in children due to the presence of red bone marrow in the long bones. Magnetic resonance imaging is used to assess the extent of lesions and whether complications are recent (edema due to recent infarction) or longstanding. Other types of MRI are used for semi-quantitative assessments of bone marrow infiltration (Quantitative Chemical Shift Imaging), but they are not available in all centers [159]. Whole-body MRI makes it possible to reduce examination time, particularly for disease monitoring purposes. The images must be carefully analyzed because additional images are sometimes required for the less visible sites, especially limb extremities (hands and feet).

Standard osseous radiographs were previously used to detect Erlenmeyer flask deformity of the femurs with widening of the lower third. This can be accompanied by thinning of the cortical bone (which may appear scalloped [160]), AVN and bone infarct sequelae (34% of cases), lytic lesions (18% of cases) that are generally well delineated without peripheral increased bone density, and the sequelae of traumatic or pathological fractures. The initial assessment should include radiological imaging of the pelvis, spine, femurs, tibiae, and humeri. Radiological imaging need not be systematically reused thereafter for monitoring purposes, except for specifically following the progression of AVN to osteoarthritis. The sensitivity of standard radiological imaging for the detection of abnormalities in GD is low [156] and the use of multiple X-rays is no longer standard practice given the limited knowledge gained from them and risk of radiation exposure.

*Bone densitometry* is used to diagnose osteopenia/osteoporosis, which is common in adults or children >5 years old, and to calculate lumbar spine and femoral bone mass. Osteopenia is defined as a T score between −1 and −2.5; osteoporosis is defined as a T score ≤ −2.5. The severity of osteopenia may be correlated with genotype, splenomegaly and hepatomegaly [86].

99mTc bone scintigraphy is sometimes used to locate bone lesions throughout the skeleton (especially the spine, femur, pelvis or tibia) when MRI is not available [161]. It enables the detection of clinically asymptomatic lesions or the sequelae of bone infarcts in atypical sites (jaws, hands or feet), as well as fractures.

Echocardiography is used to screen for pulmonary arterial hypertension.

## 9. Management

### 9.1. Usual Specific Treatments

All GD patients require regular monitoring, however specific medication is not justified in all cases. Once it has been initiated, treatment must generally be administered for life. There are currently two specific types of treatment for GD: enzyme replacement therapy (ERT) and substrate reduction therapy (SRT). The goal is to treat patients before the onset of complications, the sequelae of which are disabling or not improved by further treatment, including massive fibrous splenomegaly, AVN, secondary osteoarthritis, vertebral compression and other fractures, hepatic fibrosis and lung fibrosis.

#### 9.1.1. Enzyme Repacement Therapy

The principle of ERT is to supply the GCase lacking in the cells, particularly the Gaucher cells. After using an enzyme extracted from human placenta (alglucerase) in the early 1990s, Genzyme SA developed imiglucerase, a recombinant GCase (Cerezyme^®^, Sanofi-Genzyme). Enzymes are deglycosylated, exposing their mannose residues in order to allow their uptake by macrophage receptors and their transfer to lysosomes. Imiglucerase is produced using mammalian cells (Chinese Hamster Ovary cells); it obtained marketing authorization in 1996. Other recombinant enzymes have now been developed: velaglucerase (Vpriv^®^, Shire, authorized in 2010) produced using human fibroblasts and taliglucerase (Elelyso^®^, Pfizer) produced using carrot cells, which was available during a period of imiglucerase shortage (2009–2011), but did not obtain a marketing authorization in all countries. The differences between imiglucerase and velaglucerase are minimal. Taliglucerase undergoes specific glycosylation, related to its production in plant cells.

These products are administered intravenously. Dose and administration frequency vary depending on the country [162], often with individual adjustments of dosages. For children and “at risk adults”, a starting dose of 60 U/kg every other week (EOW) has been recommended [163]. After the achievement of therapeutic goals [164] this may be reduced to not less than 30 U/kg EOW to prevent worsening skeletal involvement during long-term maintenance therapy [163]. Some studies have reported good outcomes with low-dose high-frequency protocols, consisting of 15–30 U/kg/month given in thrice weekly doses [165,166]. Smaller total doses can decrease the cost of therapy and can be considered for patients with stable GD1 [167,168]; however, 15 U/kg EOW or lower doses may compromise skeletal response in some patients [168,169].

For many years, there was considerable debate about the optimal ERT dose, but dose–response relationships were demonstrated for hemoglobin and platelet levels, and for hepatic and splenic volumes [170]. To fully appreciate how an individual is affected by GD and how that individual is responding to treatment, it is necessary to evaluate all aspects of the disease: blood counts, organ volumes, quality of life, bone pain and crises, bone remodeling, marrow fat and bone mineral density. Assessment of growth in children, with reference to both their age- and sex-matched cohort and their mid-parental height, is also very important [171]. The dose, and less frequently the interval, of infusions can be adjusted according to the clinical course and biomarkers. Thrombocytopenia usually improves with ERT, but it may persist in individuals with residual splenomegaly and/or the presence of splenic nodules [172]. Individuals with type-1 GD report improved health-related quality of life after 24–48 months of ERT [173,174,175]. Bone marrow infiltration and osteopenia regress gradually with ERT [176]; bone pain improves, there are fewer bone crises [177], and occurrences of skeletal events decrease [178], although ERT does not completely prevent them [6]. Early treatment with ERT reduces the risk of AVN [179]. Disease control with low doses remains poorly understood but may be related to specific intracellular pharmacokinetics [120]. There are currently no criteria for the preferential use of one or other of the enzyme replacement therapies (imiglucerase or velaglucerase) to treat GD1; imiglucerase is the only ERT with a marketing authorization for GD3. None of the ERTs are indicated for GD2 as treatment has no impact on the rapid progression of its severe neurological symptoms [116]. There is no evidence that ERT has reversed, stabilized, or slowed the progression of neurological involvement [180].

Specific treatment with ERT should be considered for all GD3 patients, but only for those GD1 patients who have symptomatic clinical or biological abnormalities [163].

Safety is generally good: From 2% to 14% of patients (depending on the product) develop antibodies against the enzyme, usually without clinical signs. Allergic reactions are rare (<1.5% of patients) and include urticaria, diarrhea, hypotension or laryngeal discomfort. The risk of allergy seems a little more common with taliglucerase.

Pregnancy is not a contraindication to imiglucerase replacement therapy since no fetal malformations have been described in pregnant women for whom treatment continued. Velaglucerase also appears to be well tolerated [181]. Indeed, ERT may be required, firstly to control the disease, since GD can worsen during pregnancy, and secondly to limit thrombocytopenia which can be harmful during pregnancy or childbirth and contraindicates epidural anesthesia.

#### 9.1.2. Substrate Reduction Therapy

The aim of substrate reduction therapy (SRT) is to reduce excess cell GlcCer by decreasing its production. Miglustat (Zavesca^®^, Actelion) is a GlcCer synthase inhibitor which reduces the biosynthesis of GlcCer in Gaucher cells. It obtained a European marketing authorization in November 2002 for the treatment of mild to moderate GD1 when ERT is not suitable [182]. Miglustat is effective on the size of the liver and spleen as well as on the decrease of chitotriosidase levels, however its efficacy on hematological parameters is more limited and improvement takes longer (improvement of anemia after 24 months, little improvement of thrombocytopenia). Its efficacy on bone symptoms remains poorly evaluated.

Miglustat is administered orally at the recommended dose of 100 mg, three times daily, but doses may need to be progressively increased at treatment initiation to improve tolerance. Miglustat can produce side effects (diarrhea, weight loss, hand tremors or possible peripheral neuropathy) although these generally regress with dose reduction or treatment discontinuation. Diarrhea can be effectively controlled with loperamide and certain dietary measures (limiting the consumption of disaccharides in the form of sugars and milk) [183].

Miglustat is a second-line treatment to be used when ERT is no longer accepted by the patient or cannot be used due to intolerance. It is strictly contraindicated during pregnancy and contraceptive methods must be used by both male and female patients. To date, miglustat has not been found to have any effect on neurological symptoms in GD3, despite the fact that it crosses the blood–brain barrier.

Another substrate inhibitor, eliglustat (Cerdelga^®^, Sanofi-Genzyme) was granted a marketing authorization in 2015. It is also an orally administered GlcCer synthase inhibitor, but is more specific and more potent than miglustat, because it is an analogue of the ceramide part. It was evaluated in phase-1, -2 and -3 clinical studies comprising nearly 400 patients overall whose follow-up results were published after four years [184,185,186,187]. The studies demonstrated significant efficacy versus placebo, non-inferiority to imiglucerase (the reference product) over a two-year period and satisfactory safety. The four-year extension phase of the phase-2 study also demonstrated that it had an effect on bone [188]. This drug is suggested as first-line treatment for patients with GD1. Due to potential drug–drug interactions, its use with CYP2D6 inhibitors calls for special caution, depending on the patient’s metabolizer status (CYP2D6 genotyping required before any prescription). Eliglustat is indicated for the long-term treatment of adults with GD1 who are cytochrome 2D6-poor, intermediate or extensive metabolizers (determined previously). It is not indicated for use in ultra-extensive metabolizers. Eliglustat is not recommended in patients with pre-existing cardiac disease (e.g., congestive heart failure, recent acute myocardial infarction, bradycardia, heart block, ventricular arrhythmia, long QT syndrome), and in concomitant use with Class 1A and Class III antiarrhythmics. Adverse effects are uncommon and usually mild, including headache and pain in limb extremities in less than 10% of cases. Given that it is metabolized by cytochrome P450, certain drug–drug interactions should be anticipated. Eliglustat offers eligible patients a daily oral therapy alternative to biweekly infusions of ERT [189].

### 9.2. Other Specific Treatments

Ideally, bone marrow transplantation could cure patients with GD [190], but this treatment is no longer offered given its low benefit/risk ratio and the current availability of effective, well-tolerated therapies.

#### 9.2.1. Gene Therapy

A preliminary gene transfer protocol was used on GD3 patients [191], with the aim of introducing the *GBA1* gene into hematopoietic cells and then injecting the corrected cells into patients. Results were disappointing as the GCase levels proved too low for any clinical effect. Lentiviral vector gene transfer techniques have been used in mouse models of GD with promising results, but this approach is still at the basic research stage [192].

#### 9.2.2. Molecular Chaperones

Molecular chaperones are small molecules that enable proteins to take on the specific molecular configuration which determines their functional efficacy. They also protect proteins by preventing inappropriate aggregation, that facilitate their passage through the cell membranes and thus their transport into lysosomes, when dealing with lysosomal enzymes. Molecular chaperones can therefore help the production of functional enzymes and thus even restore the intracellular activity of mutant GCase [193]. The development of this type of treatment for GD is still in the early stages, and clinical trials have yet to be conducted, although the strategy is under consideration [194]. The effect is thought to be responsible for the positive results of pilot studies with ambroxol [195,196].

### 9.3. Symptomatic Treatments

In the era of ERT, splenectomy should be avoided in GD patients. The potential consequences of splenectomy include the usual risks of infection, thrombosis or neoplasia [197] as well as a risk of worsening the GD [198] due to an increased risk of skeletal-related events, hepatic fibrosis, cirrhosis, hepatic carcinoma and pulmonary hypertension. Splenectomy should only be performed in exceptional circumstances and considered only in rare cases of non-response to well conducted ERT with persistent severe cytopenia (usually related to massive nodular and fibrous splenomegaly) or in cases of splenic rupture. The usual recommendations for splenectomy should be followed (pre-vaccination and antibiotic prophylaxis).

Painful bone crises often require temporary immobilization and use of level I and II analgesics, sometimes even level III. Specific treatment typically reduces the frequency and intensity of these crises [199].

The use of bisphosphonates is controversial in GD because the pathophysiology of bone mass decline remains poorly understood. It appears to be the consequence of either an osteoclast or osteoblast disorder or, more likely, of an osteoblast–osteoclast coupling disorder. Specific therapy remains the best treatment for GD-related osteopenia and osteoporosis. Bisphosphonates are nonetheless often indicated in cases of persistent osteoporosis, especially in postmenopausal women. They are contraindicated during childbearing age [198].

Orthopedic surgery may be required for bone complications including AVN and pathological fractures. Except in the context of an emergency, it is preferable to operate on patients after a correction of their laboratory parameters, particularly thrombocytopenia.

Liver transplantation may be proposed for the rare patients presenting with severe liver disease progressing to fibrosis and liver failure.

To prevent bleeding, GD patients should be evaluated for coagulation abnormalities, especially prior to surgery, dental and obstetric procedures.

Psychological support should be routinely offered to GD patients and they should be put in touch with patients’ associations.

## 10. Monitoring

Patient monitoring includes regular clinical, biological and radiological evaluations. ERT improves hematological abnormalities and quality of life within a few months [200]. Biomarker levels (chitotriosidase, CCL18 and ferritin) decrease relatively quickly with ERT, prior to the normalization of platelet and hemoglobin levels [201]. Hepatosplenomegaly decreases more slowly, usually over a period of two years. Improvement of bone abnormalities is usually observed after 2–4 years of treatment, but some abnormalities remain irreversible (hepatic or splenic fibrosis, AVN and bone infarction sequelae, etc.). A significant proportion of patients show improvement, but without normalization of their cytopenia or organomegaly [202]. GD3 patients require additional neurological monitoring.

Pediatric patients are monitored more frequently: a clinical examination and full battery of laboratory tests must be carried out every six months and imaging is used as a function of disease progression.

## 11. Prognosis

Currently, available treatments make it possible to reduce cytopenias and organomegalies and to significantly decrease bone manifestations, considerably improving a patient’s quality of life.

Outcomes may be unfavorable due to aggressive, irreversible and disabling bone disease, despite specific treatment (bone events can occur despite treatment in some patients); due to the onset of Parkinson’s disease and Lewy body dementia; or the occurrence of a blood disease or cancer (hepatocellular carcinoma), whose relative risk appears higher in GD.

When ERT is ineffective in patients with GD3, progressive neurological deterioration has an impact on their prognosis. Moreover, GD3 patients can die suddenly [112]. The outcome is always fatal for patients with type 2 GD.

## 12. Conclusions

Although it is the most common of the lysosomal storage diseases, Gaucher disease remains rare and most cases present a gradual onset phenotype, which explains its delayed diagnosis. It is important to include GD in the diagnostic decision tree in cases of splenomegaly and/or thrombocytopenia, in order to avoid potentially harmful splenectomy.

Significant new insights into GD’s pathophysiology show that GCase deficiency has a much broader impact than the simple macrophage load that transforms them into Gaucher cells. These insights will open new pathways for the development of innovative therapeutic strategies. Eventually, drugs that can modify the neurological phenotype are expected to be developed. It is likely that more complex molecular studies will ultimately contribute to customized patient management.

The therapeutic advances of recent years, including the development of new enzymes and a new substrate inhibitor, represent significant progress, but research efforts must be maintained.

Patients with GD, including asymptomatic patients, must be monitored regularly to detect any complications in the disease’s progression.

## Abbreviations

AVN	Avascular Necrosis
ERT	Enzyme Replacement Therapy
GCase	glucocerebrosidase
GlcCer	glucosylceramide
GD	Gaucher Disease
SRT	Substrate Reduction Therapy

## References

[1] Ginzburg L., Kacher Y., Futerman A.H. (2004). The pathogenesis of glycosphingolipid storage disorders. Semin. Cell Dev. Biol..

[2] Hruska K.S., LaMarca M.E., Scott C.R., Sidransky E. (2008). Gaucher disease: Mutation and polymorphism spectrum in the glucocerebrosidase gene (GBA). Hum. Mutat..

[3] Vaccaro A.M., Motta M., Tatti M., Scarpa S., Masuelli L., Bhat M., Vanier M.T., Tylki-Szymanska A., Salvioli R. (2010). Saposin C mutations in Gaucher disease patients resulting in lysosomal lipid accumulation, saposin C deficiency, but normal prosaposin processing and sorting. Hum. Mol. Genet..

[4] Sidransky E. (2012). Gaucher disease: Insights from a rare Mendelian disorder. Discov. Med..

[5] Grabowski G.A. (2008). Phenotype, diagnosis, and treatment of Gaucher’s disease. Lancet.

[6] Stirnemann J., Vigan M., Hamroun D., Heraoui D., Rossi-Semerano L., Berger M.G., Rose C., Camou F., de Roux-Serratrice C., Grosbois B. (2012). The French Gaucher’s disease registry: Clinical characteristics, complications and treatment of 562 patients. Orphanet J. Rare Dis..

[7] Lee R.E. (1968). The fine structure of the cerebroside occurring in Gaucher’s disease. Proc. Natl. Acad. Sci. USA.

[8] Mikosch P., Hughes D. (2010). An overview on bone manifestations in Gaucher disease. Wiener Med. Wochenschr..

[9] Orvisky E., Park J.K., LaMarca M.E., Ginns E.I., Martin B.M., Tayebi N., Sidransky E. (2002). Glucosylsphingosine accumulation in tissues from patients with Gaucher disease: Correlation with phenotype and genotype. Mol. Genet. Metab..

[10] Kinghorn K.J., Gronke S., Castillo-Quan J.I., Woodling N.S., Li L., Sirka E., Gegg M., Mills K., Hardy J., Bjedov I. (2016). A Drosophila model of neuronopathic Gaucher disease demonstrates lysosomal-autophagic defects and altered mTOR signalling and is functionally rescued by rapamycin. J. Neurosci..

[11] Boven L.A., van Meurs M., Boot R.G., Mehta A., Boon L., Aerts J.M., Laman J.D. (2004). Gaucher cells demonstrate a distinct macrophage phenotype and resemble alternatively activated macrophages. Am. J. Clin. Pathol..

[12] Mucci J.M., Cuello M.F., Kisinovsky I., Larroude M., Delpino M.V., Rozenfeld P.A. (2015). Proinflammatory and proosteoclastogenic potential of peripheral blood mononuclear cells from Gaucher patients: Implication for bone pathology. Blood Cells Mol. Dis..

[13] Aflaki E., Moaven N., Borger D.K., Lopez G., Westbroek W., Chae J.J., Marugan J., Patnaik S., Maniwang E., Gonzalez A.N. (2016). Lysosomal storage and impaired autophagy lead to inflammasome activation in Gaucher macrophages. Aging Cell.

[14] Allen M.J., Myer B.J., Khokher A.M., Rushton N., Cox T.M. (1997). Pro-inflammatory cytokines and the pathogenesis of Gaucher’s disease: Increased release of interleukin-6 and interleukin-10. Mon. J. Assoc. Phys..

[15] Barak V., Acker M., Nisman B., Kalickman I., Abrahamov A., Zimran A., Yatziv S. (1999). Cytokines in Gaucher’s disease. Eur. Cytokine Netw..

[16] Hollak C.E., Evers L., Aerts J.M., van Oers M.H. (1997). Elevated levels of M-CSF, sCD14 and IL8 in type 1 Gaucher disease. Blood Cells Mol. Dis..

[17] van Breemen M.J., de Fost M., Voerman J.S., Laman J.D., Boot R.G., Maas M., Hollak C.E., Aerts J.M., Rezaee F. (2007). Increased plasma macrophage inflammatory protein (MIP)-1α and MIP-1β levels in type 1 Gaucher disease. Biochim. Biophys. Acta.

[18] Mistry P.K., Liu J., Yang M., Nottoli T., McGrath J., Jain D., Zhang K., Keutzer J., Chuang W.L., Mehal W.Z. (2010). Glucocerebrosidase gene-deficient mouse recapitulates Gaucher disease displaying cellular and molecular dysregulation beyond the macrophage. Proc. Natl. Acad. Sci. USA.

[19] Dekker N., van Dussen L., Hollak C.E., Overkleeft H., Scheij S., Ghauharali K., van Breemen M.J., Ferraz M.J., Groener J.E., Maas M. (2011). Elevated plasma glucosylsphingosine in Gaucher disease: Relation to phenotype, storage cell markers, and therapeutic response. Blood.

[20] Mistry P.K., Liu J., Sun L., Chuang W.L., Yuen T., Yang R., Lu P., Zhang K., Li J., Keutzer J. (2014). Glucocerebrosidase 2 gene deletion rescues type 1 Gaucher disease. Proc. Natl. Acad. Sci. USA.

[21] Hong Y.B., Kim E.Y., Jung S.C. (2004). Down-regulation of Bcl-2 in the fetal brain of the Gaucher disease mouse model: A possible role in the neuronal loss. J. Hum. Genet..

[22] Rolfs A., Giese A.K., Grittner U., Mascher D., Elstein D., Zimran A., Bottcher T., Lukas J., Hubner R., Golnitz U. (2013). Glucosylsphingosine is a highly sensitive and specific biomarker for primary diagnostic and follow-up monitoring in Gaucher disease in a non-Jewish, Caucasian cohort of Gaucher disease patients. PLoS ONE.

[23] Matloubian M., Lo C.G., Cinamon G., Lesneski M.J., Xu Y., Brinkmann V., Allende M.L., Proia R.L., Cyster J.G. (2004). Lymphocyte egress from thymus and peripheral lymphoid organs is dependent on S1P receptor 1. Nature.

[24] Lecourt S., Vanneaux V., Cras A., Freida D., Heraoui D., Herbi L., Caillaud C., Chomienne C., Marolleau J.P., Belmatoug N. (2012). Bone marrow microenvironment in an in vitro model of Gaucher disease: Consequences of glucocerebrosidase deficiency. Stem Cells Dev..

[25] Campeau P.M., Rafei M., Boivin M.N., Sun Y., Grabowski G.A., Galipeau J. (2009). Characterization of Gaucher disease bone marrow mesenchymal stromal cells reveals an altered inflammatory secretome. Blood.

[26] Berger J., Lecourt S., Vanneaux V., Rapatel C., Boisgard S., Caillaud C., Boiret-Dupre N., Chomienne C., Marolleau J.P., Larghero J. (2010). Glucocerebrosidase deficiency dramatically impairs human bone marrow haematopoiesis in an in vitro model of Gaucher disease. Br. J. Haematol..

[27] Taddei T.H., Dziura J., Chen S., Yang R., Hyogo H., Sullards C., Cohen D.E., Pastores G., Mistry P.K. (2010). High incidence of cholesterol gallstone disease in type 1 Gaucher disease: Characterizing the biliary phenotype of type 1 Gaucher disease. J. Inherit. Metab. Dis..

[28] Ron I., Horowitz M. (2005). ER retention and degradation as the molecular basis underlying Gaucher disease heterogeneity. Hum. Mol. Genet..

[29] Yang C., Wang H., Zhu D., Hong C.S., Dmitriev P., Zhang C., Li Y., Ikejiri B., Brady R.O., Zhuang Z. (2015). Mutant glucocerebrosidase in Gaucher disease recruits Hsp27 to the Hsp90 chaperone complex for proteasomal degradation. Proc. Natl. Acad. Sci. USA.

[30] Reczek D., Schwake M., Schroder J., Hughes H., Blanz J., Jin X., Brondyk W., Van Patten S., Edmunds T., Saftig P. (2007). LIMP-2 is a receptor for lysosomal mannose-6-phosphate-independent targeting of β-glucocerebrosidase. Cell.

[31] Berkovic S.F., Dibbens L.M., Oshlack A., Silver J.D., Katerelos M., Vears D.F., Lullmann-Rauch R., Blanz J., Zhang K.W., Stankovich J. (2008). Array-based gene discovery with three unrelated subjects shows SCARB2/LIMP-2 deficiency causes myoclonus epilepsy and glomerulosclerosis. Am. J. Hum. Genet..

[32] Schroen B., Leenders J.J., van Erk A., Bertrand A.T., van Loon M., van Leeuwen R.E., Kubben N., Duisters R.F., Schellings M.W., Janssen B.J. (2007). Lysosomal integral membrane protein 2 is a novel component of the cardiac intercalated disc and vital for load-induced cardiac myocyte hypertrophy. J. Exp. Med..

[33] Velayati A., DePaolo J., Gupta N., Choi J.H., Moaven N., Westbroek W., Goker-Alpan O., Goldin E., Stubblefield B.K., Kolodny E. (2011). A mutation in SCARB2 is a modifier in Gaucher disease. Hum. Mutat..

[34] Jian J., Zhao S., Tian Q.Y., Liu H., Zhao Y., Chen W.C., Grunig G., Torres P.A., Wang B.C., Zeng B. (2016). Association between progranulin and Gaucher disease. EBioMedicine.

[35] Jian J., Tian Q.Y., Hettinghouse A., Zhao S., Liu H., Wei J., Grunig G., Zhang W., Setchell K.D., Sun Y. (2016). Progranulin recruits HSP70 to β-Glucocerebrosidase and is therapeutic against Gaucher disease. EBioMedicine.

[36] Petkau T.L., Leavitt B.R. (2014). Progranulin in neurodegenerative disease. Trends Neurosci..

[37] Biegstraaten M., van Schaik I.N., Aerts J.M., Langeveld M., Mannens M.M., Bour L.J., Sidransky E., Tayebi N., Fitzgibbon E., Hollak C.E. (2011). A monozygotic twin pair with highly discordant Gaucher phenotypes. Blood Cells Mol. Dis..

[38] Elstein D., Gellman A., Altarescu G., Abrahamov A., Hadas-Halpern I., Phillips M., Margalit M., Lebel E., Itzchaki M., Zimran A. (2010). Disease severity in sibling pairs with type 1 Gaucher disease. J. Inherit. Metab. Dis..

[39] Gonzalez A., Valeiras M., Sidransky E., Tayebi N. (2014). Lysosomal integral membrane protein-2: A new player in lysosome-related pathology. Mol. Genet. Metab..

[40] Aharon-Peretz J., Badarny S., Rosenbaum H., Gershoni-Baruch R. (2005). Mutations in the glucocerebrosidase gene and Parkinson disease: Phenotype-genotype correlation. Neurology.

[41] Clark L.N., Ross B.M., Wang Y., Mejia-Santana H., Harris J., Louis E.D., Cote L.J., Andrews H., Fahn S., Waters C. (2007). Mutations in the glucocerebrosidase gene are associated with early-onset Parkinson disease. Neurology.

[42] Sato C., Morgan A., Lang A.E., Salehi-Rad S., Kawarai T., Meng Y., Ray P.N., Farrer L.A., St George-Hyslop P., Rogaeva E. (2005). Analysis of the glucocerebrosidase gene in Parkinson’s disease. Mov. Disord..

[43] Sidransky E., Nalls M.A., Aasly J.O., Aharon-Peretz J., Annesi G., Barbosa E.R., Bar-Shira A., Berg D., Bras J., Brice A. (2009). Multicenter analysis of glucocerebrosidase mutations in Parkinson’s disease. N. Engl. J. Med..

[44] Lesage S., Patin E., Condroyer C., Leutenegger A.L., Lohmann E., Giladi N., Bar-Shira A., Belarbi S., Hecham N., Pollak P. (2010). Parkinson’s disease-related LRRK2 G2019S mutation results from independent mutational events in humans. Hum. Mol. Genet..

[45] Liu G., Boot B., Locascio J.J., Jansen I.E., Winder-Rhodes S., Eberly S., Elbaz A., Brice A., Ravina B., van Hilten J.J. (2016). Neuropathic Gaucher’s mutations accelerate cognitive decline in Parkinson’s. Ann. Neurol..

[46] Cilia R., Tunesi S., Marotta G., Cereda E., Siri C., Tesei S., Zecchinelli A.L., Canesi M., Mariani C.B., Meucci N. (2016). Survival and dementia in GBA-associated Parkinson’s disease: The mutation matters. Ann. Neurol..

[47] Mazzulli J.R., Xu Y.H., Sun Y., Knight A.L., McLean P.J., Caldwell G.A., Sidransky E., Grabowski G.A., Krainc D. (2011). Gaucher disease glucocerebrosidase and α-synuclein form a bidirectional pathogenic loop in synucleinopathies. Cell.

[48] Yap T.L., Gruschus J.M., Velayati A., Westbroek W., Goldin E., Moaven N., Sidransky E., Lee J.C. (2011). α-synuclein interacts with Glucocerebrosidase providing a molecular link between Parkinson and Gaucher diseases. J. Biol. Chem..

[49] Murphy K.E., Gysbers A.M., Abbott S.K., Tayebi N., Kim W.S., Sidransky E., Cooper A., Garner B., Halliday G.M. (2014). Reduced glucocerebrosidase is associated with increased α-synuclein in sporadic Parkinson’s disease. Brain J. Neurol..

[50] Yap T.L., Velayati A., Sidransky E., Lee J.C. (2013). Membrane-bound α-synuclein interacts with glucocerebrosidase and inhibits enzyme activity. Mol. Genet. Metab..

[51] Gruschus J.M., Jiang Z., Yap T.L., Hill S.A., Grishaev A., Piszczek G., Sidransky E., Lee J.C. (2015). Dissociation of glucocerebrosidase dimer in solution by its co-factor, saposin C. Biochem. Biophys. Res. Commun..

[52] Ron I., Rapaport D., Horowitz M. (2010). Interaction between parkin and mutant glucocerebrosidase variants: A possible link between Parkinson disease and Gaucher disease. Hum. Mol. Genet..

[53] Barkhuizen M., Anderson D.G., Grobler A.F. (2016). Advances in GBA-associated Parkinson’s disease—Pathology, presentation and therapies. Neurochem. Int..

[54] Cullen V., Sardi S.P., Ng J., Xu Y.H., Sun Y., Tomlinson J.J., Kolodziej P., Kahn I., Saftig P., Woulfe J. (2011). Acid β-glucosidase mutants linked to Gaucher disease, Parkinson disease, and Lewy body dementia alterα-synuclein processing. Ann. Neurol..

[55] Yap T.L., Jiang Z., Heinrich F., Gruschus J.M., Pfefferkorn C.M., Barros M., Curtis J.E., Sidransky E., Lee J.C. (2015). Structural features of membrane-bound glucocerebrosidase and α-synuclein probed by neutron reflectometry and fluorescence spectroscopy. J. Biol. Chem..

[56] Siebert M., Sidransky E., Westbroek W. (2014). Glucocerebrosidase is shaking up the synucleinopathies. Brain J. Neurol..

[57] Horowitz M., Elstein D., Zimran A., Goker-Alpan O. (2016). New directions in Gaucher disease. Hum. Mutat..

[58] Yap T.L., Gruschus J.M., Velayati A., Sidransky E., Lee J.C. (2013). Saposin C protects glucocerebrosidase against α-synuclein inhibition. Biochemistry.

[59] Arends M., van Dussen L., Biegstraaten M., Hollak C.E. (2013). Malignancies and monoclonal gammopathy in Gaucher disease; a systematic review of the literature. Br. J. Haematol..

[60] Rosenbloom B.E., Weinreb N.J., Zimran A., Kacena K.A., Charrow J., Ward E. (2005). Gaucher disease and cancer incidence: A study from the Gaucher Registry. Blood.

[61] Taddei T.H., Kacena K.A., Yang M., Yang R., Malhotra A., Boxer M., Aleck K.A., Rennert G., Pastores G.M., Mistry P.K. (2009). The underrecognized progressive nature of N370S Gaucher disease and assessment of cancer risk in 403 patients. Am. J. Hematol..

[62] De Fost M., Vom Dahl S., Weverling G.J., Brill N., Brett S., Haussinger D., Hollak C.E. (2006). Increased incidence of cancer in adult Gaucher disease in Western Europe. Blood Cells Mol. Dis..

[63] Landgren O., Turesson I., Gridley G., Caporaso N.E. (2007). Risk of malignant disease among 1525 adult male US Veterans with Gaucher disease. Arch. Intern. Med..

[64] Astudillo L., Therville N., Colacios C., Segui B., Andrieu-Abadie N., Levade T. (2015). Glucosylceramidases and malignancies in mammals. Biochimie.

[65] Braudeau C., Graveleau J., Rimbert M., Neel A., Hamidou M., Grosbois B., Besancon A., Giraudet S., Terrien C., Josien R. (2013). Altered innate function of plasmacytoid dendritic cells restored by enzyme replacement therapy in Gaucher disease. Blood Cells Mol. Dis..

[66] Burstein Y., Zakuth V., Rechavi G., Spirer Z. (1987). Abnormalities of cellular immunity and natural killer cells in Gaucher’s disease. J. Clin. Lab. Immunol..

[67] Pavlova E.V., Wang S.Z., Archer J., Dekker N., Aerts J.M., Karlsson S., Cox T.M. (2013). B cell lymphoma and myeloma in murine Gaucher’s disease. J. Pathol..

[68] Pavlova E.V., Archer J., Wang S., Dekker N., Aerts J.M., Karlsson S., Cox T.M. (2015). Inhibition of UDP-glucosylceramide synthase in mice prevents Gaucher disease-associated B-cell malignancy. J. Pathol..

[69] Nair S., Branagan A.R., Liu J., Boddupalli C.S., Mistry P.K., Dhodapkar M.V. (2016). Clonal immunoglobulin against lysolipids in the origin of myeloma. N. Engl. J. Med..

[70] Medrano-Engay B., Irun P., Gervas-Arruga J., Andrade-Campos M., Andreu V., Alfonso P., Pocovi M., Giraldo P. (2014). Iron homeostasis and infIammatory biomarker analysis in patients with type 1 Gaucher disease. Blood Cells Mol. Dis..

[71] Stirnemann J., Boutten A., Vincent C., Mekinian A., Heraoui D., Fantin B., Fain O., Mentre F., Belmatoug N. (2011). Impact of imiglucerase on the serum glycosylated-ferritin level in Gaucher disease. Blood Cells Mol. Dis..

[72] Stirnemann J., Belmatoug N., Vincent C., Fain O., Fantin B., Mentre F. (2010). Bone events and evolution of biologic markers in Gaucher disease before and during treatment. Arthritis Res. Ther..

[73] Sidransky E. (2004). Gaucher disease: Complexity in a “simple” disorder. Mol. Genet. Metab..

[74] Charrow J., Andersson H.C., Kaplan P., Kolodny E.H., Mistry P., Pastores G., Rosenbloom B.E., Scott C.R., Wappner R.S., Weinreb N.J. (2000). The Gaucher registry: Demographics and disease characteristics of 1698 patients with Gaucher disease. Arch. Intern. Med..

[75] Kaplan P., Andersson H.C., Kacena K.A., Yee J.D. (2006). The clinical and demographic characteristics of nonneuronopathic Gaucher disease in 887 children at diagnosis. Arch. Pediatrics Adolesc. Med..

[76] Grabowski G.A., Zimran A., Ida H. (2015). Gaucher disease types 1 and 3: Phenotypic characterization of large populations from the ICGG Gaucher Registry. Am. J. Hematol..

[77] Hill S.C., Reinig J.W., Barranger J.A., Fink J., Shawker T.H. (1986). Gaucher disease: Sonographic appearance of the spleen. Radiology.

[78] Neudorfer O., Hadas-Halpern I., Elstein D., Abrahamov A., Zimran A. (1997). Abdominal ultrasound findings mimicking hematological malignancies in a study of 218 Gaucher patients. Am. J. Hematol..

[79] Regenboog M., Bohte A.E., Somers I., van Delden O.M., Maas M., Hollak C.E. (2016). Imaging characteristics of focal splenic and hepatic lesions in type 1 Gaucher disease. Blood Cells Mol. Dis..

[80] Rosenbaum H. (2014). Hemorrhagic aspects of Gaucher disease. Rambam Maimonides Med. J..

[81] Gillis S., Hyam E., Abrahamov A., Elstein D., Zimran A. (1999). Platelet function abnormalities in Gaucher disease patients. Am. J. Hematol..

[82] Wenstrup R.J., Roca-Espiau M., Weinreb N.J., Bembi B. (2002). Skeletal aspects of Gaucher disease: A review. Br. J. Radiol..

[83] Clarke L.A., Hollak C.E. (2015). The clinical spectrum and pathophysiology of skeletal complications in lysosomal storage disorders. Best Pract. Res. Clin. Endocrinol. Metab..

[84] Marcucci G., Zimran A., Bembi B., Kanis J., Reginster J.Y., Rizzoli R., Cooper C., Brandi M.L. (2014). Gaucher disease and bone manifestations. Calcif. Tissue Int..

[85] Yossipovitch Z.H., Herman G., Makin M. (1965). Aseptic osteomyelitis in Gaucher’s disease. Isr. J. Med. Sci..

[86] Pastores G.M., Wallenstein S., Desnick R.J., Luckey M.M. (1996). Bone density in Type 1 Gaucher disease. J. Bone Miner. Res. Off. J. Am. Soc. Bone Miner. Res..

[87] Deegan P.B., Pavlova E., Tindall J., Stein P.E., Bearcroft P., Mehta A., Hughes D., Wraith J.E., Cox T.M. (2011). Osseous manifestations of adult Gaucher disease in the era of enzyme replacement therapy. Medicine.

[88] Poll L.W., Koch J.A., vom Dahl S., Loxtermann E., Sarbia M., Niederau C., Haussinger D., Modder U. (2000). Extraosseous manifestation of Gaucher’s disease type I: MR and histological appearance. Eur. Radiol..

[89] Zver S., Bracko M., Andoljsek D. (2010). Primary bone angiosarcoma in a patient with Gaucher disease. Int. J. Hematol..

[90] Kenan S., Abdelwahab I.F., Hermann G., Klein M., Pastores G. (1996). Osteoblastoma of the humerus associated with type-I Gaucher’s disease. A case report. J. Bone Joint Surg. Br. Vol..

[91] Bembi B., Ciana G., Mengel E., Terk M.R., Martini C., Wenstrup R.J. (2002). Bone complications in children with Gaucher disease. Br. J. Radiol..

[92] Faden M.A., Krakow D., Ezgu F., Rimoin D.L., Lachman R.S. (2009). The Erlenmeyer flask bone deformity in the skeletal dysplasias. Am. J. Med. Genet. Part A.

[93] Amir G., Ron N. (1999). Pulmonary pathology in Gaucher’s disease. Hum. Pathol..

[94] Mistry P.K., Sirrs S., Chan A., Pritzker M.R., Duffy T.P., Grace M.E., Meeker D.P., Goldman M.E. (2002). Pulmonary hypertension in type 1 Gaucher’s disease: Genetic and epigenetic determinants of phenotype and response to therapy. Mol. Genet. Metab..

[95] Santamaria F., Parenti G., Guidi G., Filocamo M., Strisciuglio P., Grillo G., Farina V., Sarnelli P., Rizzolo M.G., Rotondo A. (1998). Pulmonary manifestations of Gaucher disease: An increased risk for L444P homozygotes?. Am. J. Respir. Critic. Care Med..

[96] Santoro D., Rosenbloom B.E., Cohen A.H. (2002). Gaucher disease with nephrotic syndrome: Response to enzyme replacement therapy. Am. J. Kidney Dis..

[97] Goldblatt J., Beighton P. (1984). Cutaneous manifestations of Gaucher disease. Br. J. Dermatol..

[98] Raz J., Anteby I., Livni N., Benezra D. (1993). Chronic uveitis in Gaucher’s disease. Ocular Immunol. Inflamm..

[99] Bruscolini A., Pirraglia M.P., Restivo L., Spinucci G., Abbouda A. (2012). A branch retinal artery occlusion in a patient with Gaucher disease. Graefes Arch. Clin. Exp. Ophthalmol..

[100] Petrohelos M., Tricoulis D., Kotsiras I., Vouzoukos A. (1975). Ocular manifestations of Gaucher’s disease. Am. J. Ophthalmol..

[101] Roghi A., Poggiali E., Cassinerio E., Pedrotti P., Giuditta M., Milazzo A., Quattrocchi G., Cappellini M.D. (2016). The role of cardiac magnetic resonance to assess the cardiac involvement in Gaucher type 1 patients: Morphological and functional evaluations. J. Cardiovasc. Med..

[102] Langeveld M., de Fost M., Aerts J.M., Sauerwein H.P., Hollak C.E. (2008). Overweight, insulin resistance and type II diabetes in type I Gaucher disease patients in relation to enzyme replacement therapy. Blood Cells Mol. Dis..

[103] Elstein D., Rosenmann E., Reinus C., Paz J., Altarescu G., Zimran A. (2003). Amyloidosis and gastric bleeding in a patient with Gaucher disease. J. Clin. Gastroenterol..

[104] Hrebicek M., Zeman J., Musilova J., Hodanova K., Renkema G.H., Veprekova L., Ledvinova J., Hrebicek D., Sokolova J., Aerts J.M. (1996). A case of type I Gaucher disease with cardiopulmonary amyloidosis and chitotriosidase deficiency. Virchows Arch. Int. J. Pathol..

[105] Hanash S.M., Rucknagel D.L., Heidelberger K.P., Radin N.S. (1978). Primary amyloidosis associated with Gaucher’s disease. Ann. Intern. Med..

[106] Alcalay R.N., Dinur T., Quinn T., Sakanaka K., Levy O., Waters C., Fahn S., Dorovski T., Chung W.K., Pauciulo M. (2014). Comparison of Parkinson risk in Ashkenazi Jewish patients with Gaucher disease and GBA heterozygotes. JAMA Neurol..

[107] Bultron G., Kacena K., Pearson D., Boxer M., Yang R., Sathe S., Pastores G., Mistry P.K. (2010). The risk of Parkinson’s disease in type 1 Gaucher disease. J. Inherit. Metab. Dis..

[108] Biegstraaten M., Mengel E., Marodi L., Petakov M., Niederau C., Giraldo P., Hughes D., Mrsic M., Mehta A., Hollak C.E. (2010). Peripheral neuropathy in adult type 1 Gaucher disease: A 2-year prospective observational study. Brain J. Neurol..

[109] Tajima A., Yokoi T., Ariga M., Ito T., Kaneshiro E., Eto Y., Ida H. (2009). Clinical and genetic study of Japanese patients with type 3 Gaucher disease. Mol. Genet. Metab..

[110] Tylki-Szymanska A., Vellodi A., El-Beshlawy A., Cole J.A., Kolodny E. (2010). Neuronopathic Gaucher disease: Demographic and clinical features of 131 patients enrolled in the International Collaborative Gaucher Group Neurological Outcomes Subregistry. J. Inherit. Metab. Dis..

[111] Kraoua I., Sedel F., Caillaud C., Froissart R., Stirnemann J., Chaurand G., Flodrops H., Tari S., Gourfinkel-An I., Mathieu S. (2011). A French experience of type 3 Gaucher disease: Phenotypic diversity and neurological outcome of 10 patients. Brain Dev..

[112] Abdelwahab M., Blankenship D., Schiffmann R. (2016). Long-term follow-up and sudden unexpected death in Gaucher disease type 3 in Egypt. Neurol. Genet..

[113] George R., McMahon J., Lytle B., Clark B., Lichtin A. (2001). Severe valvular and aortic arch calcification in a patient with Gaucher’s disease homozygous for the D409H mutation. Clin. Genet..

[114] Cindik N., Ozcay F., Suren D., Akkoyun I., Gokdemir M., Varan B., Alehan F., Ozbek N., Tokel K. (2010). Gaucher disease with communicating hydrocephalus and cardiac involvement. Clin. Cardiol..

[115] Tamargo R.J., Velayati A., Goldin E., Sidransky E. (2012). The role of saposin C in Gaucher disease. Mol. Genet. Metab..

[116] Mignot C., Doummar D., Maire I., De Villemeur T.B., French Type 2 Gaucher Disease Study Group (2006). Type 2 Gaucher disease: 15 new cases and review of the literature. Brain Dev..

[117] Mignot C., Gelot A., De Villemeur T.B. (2013). Gaucher disease. Handb. Clin. Neurol..

[118] Mignot C., Gelot A., Bessieres B., Daffos F., Voyer M., Menez F., Fallet Bianco C., Odent S., Le Duff D., Loget P. (2003). Perinatal-lethal Gaucher disease. Am. J. Med. Genet. Part A.

[119] Neufeld E.F. (1991). Lysosomal storage diseases. Annu. Rev. Biochem..

[120] Berger J., Stirnemann J., Bourgne C., Pereira B., Pigeon P., Heraoui D., Froissart R., Rapatel C., Rose C., Belmatoug N. (2012). The uptake of recombinant glucocerebrosidases by blood monocytes from type 1 Gaucher disease patients is variable. Br. J. Haematol..

[121] Costello R., O’Callaghan T., Sebahoun G. (2006). Gaucher disease and multiple myeloma. Leukemia Lymphoma.

[122] Robak T., Urbanska-Rys H., Jerzmanowski P., Bartkowiak J., Liberski P., Kordek R. (2002). Lymphoplasmacytic lymphoma with monoclonal gammopathy-related pseudo-Gaucher cell infiltration in bone marrow and spleen—Diagnostic and therapeutic dilemmas. Leukemia Lymphoma.

[123] Yang H.S., Cho K.S., Park T.S. (2013). Chronic myeloid leukemia with marked splenomegaly and pseudo-Gaucher cells. Blood Res..

[124] Stewart A.J., Jones R.D. (1999). Pseudo-Gaucher cells in myelodysplasia. J. Clin. Pathol..

[125] Busarla S.V., Sadruddin F.A., Sohani A.R. (2013). Pseudo-Gaucher cells in disseminated mycobacterial infection. Am. J. Hematol..

[126] Horowitz M., Wilder S., Horowitz Z., Reiner O., Gelbart T., Beutler E. (1989). The human glucocerebrosidase gene and pseudogene: Structure and evolution. Genomics.

[127] Koprivica V., Stone D.L., Park J.K., Callahan M., Frisch A., Cohen I.J., Tayebi N., Sidransky E. (2000). Analysis and classification of 304 mutant alleles in patients with type 1 and type 3 Gaucher disease. Am. J. Hum. Genet..

[128] Yoshida S., Kido J., Matsumoto S., Momosaki K., Mitsubuchi H., Shimazu T., Sugawara K., Endo F., Nakamura K. (2016). Prenatal diagnosis of Gaucher disease using next-generation sequencing. Pediatr. Int..

[129] Hollak C.E., Belmatoug N., Cole J.A., Vom Dahl S., Deegan P.B., Goldblatt J., Rosenbloom B., van Dussen L., Tylki-Szymanska A., Weinreb N.J. (2012). Characteristics of type I Gaucher disease associated with persistent thrombocytopenia after treatment with imiglucerase for 4–5 years. Br. J. Haematol..

[130] Mitrovic M., Elezovic I., Miljic P., Suvajdzic N. (2014). Acquired von Willebrand syndrome in patients with Gaucher disease. Blood Cells Mol. Dis..

[131] Spectre G., Roth B., Ronen G., Rosengarten D., Elstein D., Zimran A., Varon D., Revel-Vilk S. (2011). Platelet adhesion defect in type I Gaucher Disease is associated with a risk of mucosal bleeding. Br. J. Haematol..

[132] Grosbois B., Rose C., Noel E., Serratrice Cde R., Dobbelaere D., Gressin V., Cherin P., Hartmann A., Javier R.M., Clerson P. (2009). Gaucher disease and monoclonal gammopathy: A report of 17 cases and impact of therapy. Blood Cells Mol. Dis..

[133] De Fost M., Out T.A., de Wilde F.A., Tjin E.P., Pals S.T., van Oers M.H., Boot R.G., Aerts J.F., Maas M., Vom Dahl S. (2008). Immunoglobulin and free light chain abnormalities in Gaucher disease type I: Data from an adult cohort of 63 patients and review of the literature. Ann. Hematol..

[134] Brautbar A., Elstein D., Pines G., Abrahamov A., Zimran A. (2004). Effect of enzyme replacement therapy on gammopathies in Gaucher disease. Blood Cells Mol. Dis..

[135] Hollak C.E., van Weely S., van Oers M.H., Aerts J.M. (1994). Marked elevation of plasma chitotriosidase activity. A novel hallmark of Gaucher disease. J. Clin. Investig..

[136] Van Dussen L., Hendriks E.J., Groener J.E., Boot R.G., Hollak C.E., Aerts J.M. (2014). Value of plasma chitotriosidase to assess non-neuronopathic Gaucher disease severity and progression in the era of enzyme replacement therapy. J. Inher. Metab. Dis..

[137] Bussink A.P., Verhoek M., Vreede J., Ghauharali-van der Vlugt K., Donker-Koopman W.E., Sprenger R.R., Hollak C.E., Aerts J.M., Boot R.G. (2009). Common G102S polymorphism in chitotriosidase differentially affects activity towards 4-methylumbelliferyl substrates. FEBS J..

[138] Bargagli E., Bennett D., Maggiorelli C., Di Sipio P., Margollicci M., Bianchi N., Rottoli P. (2013). Human chitotriosidase: A sensitive biomarker of sarcoidosis. J. Clin. Immunol..

[139] Aguilera B., Ghauharali-van der Vlugt K., Helmond M.T., Out J.M., Donker-Koopman W.E., Groener J.E., Boot R.G., Renkema G.H., van der Marel G.A., van Boom J.H. (2003). Transglycosidase activity of chitotriosidase: Improved enzymatic assay for the human macrophage chitinase. J. Biol. Chem..

[140] Gordon S. (2003). Alternative activation of macrophages. Nat. Rev. Immunol..

[141] Islam S.A., Ling M.F., Leung J., Shreffler W.G., Luster A.D. (2013). Identification of human CCR8 as a CCL18 receptor. J. Exp. Med..

[142] Bonella F., Costabel U. (2014). Biomarkers in connective tissue disease-associated interstitial lung disease. Semin. Respir. Crit. Care Med..

[143] Tsicopoulos A., Chang Y., Ait Yahia S., de Nadai P., Chenivesse C. (2013). Role of CCL18 in asthma and lung immunity. Clin. Exp. Allergy J. Br. Soc. Allergy Clin. Immunol..

[144] Boot R.G., Verhoek M., de Fost M., Hollak C.E., Maas M., Bleijlevens B., van Breemen M.J., van Meurs M., Boven L.A., Laman J.D. (2004). Marked elevation of the chemokine CCL18/PARC in Gaucher disease: A novel surrogate marker for assessing therapeutic intervention. Blood.

[145] Deegan P.B., Moran M.T., McFarlane I., Schofield J.P., Boot R.G., Aerts J.M., Cox T.M. (2005). Clinical evaluation of chemokine and enzymatic biomarkers of Gaucher disease. Blood Cells Mol. Dis..

[146] Fuller M., Szer J., Stark S., Fletcher J.M. (2015). Rapid, single-phase extraction of glucosylsphingosine from plasma: A universal screening and monitoring tool. Clin. Chim. Acta Int. J. Clin. Chem..

[147] Mirzaian M., Wisse P., Ferraz M.J., Gold H., Donker-Koopman W.E., Verhoek M., Overkleeft H.S., Boot R.G., Kramer G., Dekker N. (2015). Mass spectrometric quantification of glucosylsphingosine in plasma and urine of type 1 Gaucher patients using an isotope standard. Blood Cells Mol. Dis..

[148] Murugesan V., Chuang W.L., Liu J., Lischuk A., Kacena K., Lin H., Pastores G.M., Yang R., Keutzer J., Zhang K. (2016). Glucosylsphingosine is a key biomarker of Gaucher disease. Am. J. Hematol..

[149] Mekinian A., Stirnemann J., Belmatoug N., Heraoui D., Fantin B., Fain O., Charpentier A., Rose C. (2012). Ferritinemia during type 1 Gaucher disease: Mechanisms and progression under treatment. Blood Cells Mol. Dis..

[150] Stein P., Yu H., Jain D., Mistry P.K. (2010). Hyperferritinemia and iron overload in type 1 Gaucher disease. Am. J. Hematol..

[151] Aerts J.M., Kallemeijn W.W., Wegdam W., Joao Ferraz M., van Breemen M.J., Dekker N., Kramer G., Poorthuis B.J., Groener J.E., Cox-Brinkman J. (2011). Biomarkers in the diagnosis of lysosomal storage disorders: Proteins, lipids, and inhibodies. J. Inher. Metab. Dis..

[152] Hughes D., Cappellini M.D., Berger M., Van Droogenbroeck J., de Fost M., Janic D., Marinakis T., Rosenbaum H., Villarubia J., Zhukovskaya E. (2007). Recommendations for the management of the haematological and onco-haematological aspects of Gaucher disease. Br. J. Haematol..

[153] Sellos-Moura M., Barzegar S., Pan L., Shi P., Oommen S., Durant J., Ruiz J.A. (2011). Development of a panel of highly sensitive, equivalent assays for detection of antibody responses to velaglucerase alfa or imiglucerase enzyme replacement therapy in patients with Gaucher disease. J. Immunol. Methods.

[154] De Fost M., Langeveld M., Franssen R., Hutten B.A., Groener J.E., de Groot E., Mannens M.M., Bikker H., Aerts J.M., Kastelein J.J. (2009). Low HDL cholesterol levels in type I Gaucher disease do not lead to an increased risk of cardiovascular disease. Atherosclerosis.

[155] Elstein D., Tiomkin M., Hadas-Halpern I., Zimran A. (2011). Organ volume by computed tomography correlates with longitudinal axis on ultrasound in patients with Gaucher disease. Ultrasound Q..

[156] Vom Dahl S., Poll L., Di Rocco M., Ciana G., Denes C., Mariani G., Maas M. (2006). Evidence-based recommendations for monitoring bone disease and the response to enzyme replacement therapy in Gaucher patients. Curr. Med. Res. Opin..

[157] Maas M., van Kuijk C., Stoker J., Hollak C.E., Akkerman E.M., Aerts J.F., den Heeten G.J. (2003). Quantification of bone involvement in Gaucher disease: MR imaging bone marrow burden score as an alternative to Dixon quantitative chemical shift MR imaging—Initial experience. Radiology.

[158] Fedida B., Touraine S., Stirnemann J., Belmatoug N., Laredo J.D., Petrover D. (2015). Bone marrow involvement in Gaucher disease at MRI: What long-term evolution can we expect under enzyme replacement therapy?. Eur. Radiol..

[159] Hollak C., Maas M., Akkerman E., den Heeten A., Aerts H. (2001). Dixon quantitative chemical shift imaging is a sensitive tool for the evaluation of bone marrow responses to individualized doses of enzyme supplementation therapy in type 1 Gaucher disease. Blood Cells Mol. Dis..

[160] Maas M., Poll L.W., Terk M.R. (2002). Imaging and quantifying skeletal involvement in Gaucher disease. Br. J. Radiol..

[161] Mikosch P., Zitter F., Gallowitsch H.J., Wurtz F., Lind P., Mehta A.B., Hughes D.A. (2008). Bone- and bone marrow scintigraphy in Gaucher disease type 1. Nukl. Nucl. Med..

[162] Goker-Alpan O. (2011). Therapeutic approaches to bone pathology in Gaucher disease: Past, present and future. Mol. Genet. Metab..

[163] Andersson H.C., Charrow J., Kaplan P., Mistry P., Pastores G.M., Prakash-Cheng A., Rosenbloom B.E., Scott C.R., Wappner R.S., Weinreb N.J. (2005). Individualization of long-term enzyme replacement therapy for Gaucher disease. Genet. Med..

[164] Pastores G.M., Weinreb N.J., Aerts H., Andria G., Cox T.M., Giralt M., Grabowski G.A., Mistry P.K., Tylki-Szymanska A. (2004). Therapeutic goals in the treatment of Gaucher disease. Sem. Hematol..

[165] Beutler E., Demina A., Laubscher K., Garver P., Gelbart T., Balicki D., Vaughan L. (1995). The clinical course of treated and untreated Gaucher disease. A study of 45 patients. Blood Cells Mol. Dis..

[166] Zimran A., Hadas-Halpern I., Zevin S., Levy-Lahad E., Abrahamov A. (1993). Low-dose high-frequency enzyme replacement therapy for very young children with severe Gaucher disease. Br. J. Haematol..

[167] Figueroa M.L., Rosenbloom B.E., Kay A.C., Garver P., Thurston D.W., Koziol J.A., Gelbart T., Beutler E. (1992). A less costly regimen of alglucerase to treat Gaucher’s disease. N. Engl. J. Med..

[168] Wilson C., Spearing R., Teague L., Robertson P., Blacklock H. (2007). The outcome of clinical parameters in adults with severe Type I Gaucher disease using very low dose enzyme replacement therapy. Mol. Genet. Metab..

[169] Cohen I.J., Katz K., Kornreich L., Horev G., Frish A., Zaizov R. (1998). Low-dose high-frequency enzyme replacement therapy prevents fractures without complete suppression of painful bone crises in patients with severe juvenile onset type I Gaucher disease. Blood Cells Mol. Dis..

[170] Grabowski G.A., Kacena K., Cole J.A., Hollak C.E., Zhang L., Yee J., Mistry P.K., Zimran A., Charrow J., vom Dahl S. (2009). Dose-response relationships for enzyme replacement therapy with imiglucerase/alglucerase in patients with Gaucher disease type 1. Genet. Med..

[171] Charrow J., Scott C.R. (2015). Long-term treatment outcomes in Gaucher disease. Am. J. Hematol..

[172] Stein P., Malhotra A., Haims A., Pastores G.M., Mistry P.K. (2010). Focal splenic lesions in type I Gaucher disease are associated with poor platelet and splenic response to macrophage-targeted enzyme replacement therapy. J. Inherit. Metab. Dis..

[173] Damiano A.M., Pastores G.M., Ware J.E. (1998). The health-related quality of life of adults with Gaucher’s disease receiving enzyme replacement therapy: Results from a retrospective study. Qual. Life Res. Int. J. Qual. Life Asp. Treat. Care Rehabil..

[174] Masek B.J., Sims K.B., Bove C.M., Korson M.S., Short P., Norman D.K. (1999). Quality of life assessment in adults with type 1 Gaucher disease. Qual. Life Res. Int. J. Qual. Life Asp. Treatm. Care Rehabil..

[175] Weinreb N., Barranger J., Packman S., Prakash-Cheng A., Rosenbloom B., Sims K., Angell J., Skrinar A., Pastores G.M. (2007). Imiglucerase (Cerezyme) improves quality of life in patients with skeletal manifestations of Gaucher disease. Clin. Genet..

[176] Wenstrup R.J., Kacena K.A., Kaplan P., Pastores G.M., Prakash-Cheng A., Zimran A., Hangartner T.N. (2007). Effect of enzyme replacement therapy with imiglucerase on BMD in type 1 Gaucher disease. J. Bone Miner. Res..

[177] Charrow J., Dulisse B., Grabowski G.A., Weinreb N.J. (2007). The effect of enzyme replacement therapy on bone crisis and bone pain in patients with type 1 Gaucher disease. Clin. Genet..

[178] Mistry P.K., Weinreb N.J., Kaplan P., Cole J.A., Gwosdow A.R., Hangartner T. (2011). Osteopenia in Gaucher disease develops early in life: Response to imiglucerase enzyme therapy in children, adolescents and adults. Blood Cells Mol. Dis..

[179] Mistry P.K., Deegan P., Vellodi A., Cole J.A., Yeh M., Weinreb N.J. (2009). Timing of initiation of enzyme replacement therapy after diagnosis of type 1 Gaucher disease: Effect on incidence of avascular necrosis. Br. J. Haematol..

[180] Weiss K., Gonzalez A.N., Lopez G., Pedoeim L., Groden C., Sidransky E. (2015). The clinical management of Type 2 Gaucher disease. Mol. Genet. Metab..

[181] Elstein D., Hughes D., Goker-Alpan O., Stivel M., Baris H.N., Cohen I.J., Granovsky-Grisaru S., Samueloff A., Mehta A., Zimran A. (2014). Outcome of pregnancies in women receiving velaglucerase alfa for Gaucher disease. J. Obstet. Gynaecol. Res..

[182] Aerts J.M., Hollak C.E., Boot R.G., Groener J.E., Maas M. (2006). Substrate reduction therapy of glycosphingolipid storage disorders. J. Inher. Metab. Dis..

[183] Belmatoug N., Burlina A., Giraldo P., Hendriksz C.J., Kuter D.J., Mengel E., Pastores G.M. (2011). Gastrointestinal disturbances and their management in miglustat-treated patients. J. Inherit. Metab. Dis..

[184] Lukina E., Watman N., Arreguin E.A., Banikazemi M., Dragosky M., Iastrebner M., Rosenbaum H., Phillips M., Pastores G.M., Rosenthal D.I. (2010). A phase 2 study of eliglustat tartrate (Genz-112638), an oral substrate reduction therapy for Gaucher disease type 1. Blood.

[185] Lukina E., Watman N., Dragosky M., Pastores G.M., Arreguin E.A., Rosenbaum H., Zimran A., Angell J., Ross L., Puga A.C. (2014). Eliglustat, an investigational oral therapy for Gaucher disease type 1: Phase 2 trial results after 4 years of treatment. Blood Cells Mol. Dis..

[186] Cox T.M., Drelichman G., Cravo R., Balwani M., Burrow T.A., Martins A.M., Lukina E., Rosenbloom B., Ross L., Angell J. (2015). Eliglustat compared with imiglucerase in patients with Gaucher’s disease type 1 stabilised on enzyme replacement therapy: A phase 3, randomised, open-label, non-inferiority trial. Lancet.

[187] Mistry P.K., Lukina E., Ben Turkia H., Amato D., Baris H., Dasouki M., Ghosn M., Mehta A., Packman S., Pastores G. (2015). Effect of oral eliglustat on splenomegaly in patients with Gaucher disease type 1: The ENGAGE randomized clinical trial. JAMA.

[188] Kamath R.S., Lukina E., Watman N., Dragosky M., Pastores G.M., Arreguin E.A., Rosenbaum H., Zimran A., Aguzzi R., Puga A.C. (2014). Skeletal improvement in patients with Gaucher disease type 1: A phase 2 trial of oral eliglustat. Skelet. Radiol..

[189] Belmatoug N., Di Rocco M., Fraga C., Giraldo P., Hughes D., Lukina E., Maison-Blanche P., Merkel M., Niederau C., Plckinger U. (2017). Management and monitoring recommendations for the use of eliglustat in adults with type 1 Gaucher disease in Europe. Eur. J. Intern. Med..

[190] Ringden O., Groth C.G., Erikson A., Granqvist S., Mansson J.E., Sparrelid E. (1995). Ten years’ experience of bone marrow transplantation for Gaucher disease. Transplantation.

[191] Dunbar C.E., Kohn D.B., Schiffmann R., Barton N.W., Nolta J.A., Esplin J.A., Pensiero M., Long Z., Lockey C., Emmons R.V. (1998). Retroviral transfer of the glucocerebrosidase gene into CD34+ cells from patients with Gaucher disease: In vivo detection of transduced cells without myeloablation. Hum. Gene Ther..

[192] Dahl M., Doyle A., Olsson K., Mansson J.E., Marques A.R., Mirzaian M., Aerts J.M., Ehinger M., Rothe M., Modlich U. (2015). Lentiviral gene therapy using cellular promoters cures type 1 Gaucher disease in mice. Mol. Ther. J. Am. Soc. Gene Ther..

[193] Sanchez-Martinez A., Beavan M., Gegg M.E., Chau K.Y., Whitworth A.J., Schapira A.H. (2016). Parkinson disease-linked GBA mutation effects reversed by molecular chaperones in human cell and fly models. Sci. Rep..

[194] Parenti G. (2009). Treating lysosomal storage diseases with pharmacological chaperones: From concept to clinics. EMBO Mol. Med..

[195] Maegawa G.H., Tropak M.B., Buttner J.D., Rigat B.A., Fuller M., Pandit D., Tang L., Kornhaber G.J., Hamuro Y., Clarke J.T. (2009). Identification and characterization of ambroxol as an enzyme enhancement agent for Gaucher disease. J. Biol. Chem..

[196] McNeill A., Magalhaes J., Shen C., Chau K.Y., Hughes D., Mehta A., Foltynie T., Cooper J.M., Abramov A.Y., Gegg M. (2014). Ambroxol improves lysosomal biochemistry in glucocerebrosidase mutation-linked Parkinson disease cells. Brain J. Neurol..

[197] Kristinsson S.Y., Gridley G., Hoover R.N., Check D., Landgren O. (2014). Long-term risks after splenectomy among 8,149 cancer-free American veterans: A cohort study with up to 27 years follow-up. Haematologica.

[198] Cox T.M., Aerts J.M., Belmatoug N., Cappellini M.D., vom Dahl S., Goldblatt J., Grabowski G.A., Hollak C.E., Hwu P., Maas M. (2008). Management of non-neuronopathic Gaucher disease with special reference to pregnancy, splenectomy, bisphosphonate therapy, use of biomarkers and bone disease monitoring. J. Inherit. Metab. Dis..

[199] Baris H.N., Weisz Hubshman M., Bar-Sever Z., Kornreich L., Shkalim Zemer V., Cohen I.J. (2015). Re-evaluation of bone pain in patients with type 1 Gaucher disease suggests that bone crises occur in small bones as well as long bones. Blood Cells Mol. Dis..

[200] Barton N.W., Brady R.O., Dambrosia J.M., Di Bisceglie A.M., Doppelt S.H., Hill S.C., Mankin H.J., Murray G.J., Parker R.I., Argoff C.E. (1991). Replacement therapy for inherited enzyme deficiency—Macrophage-targeted glucocerebrosidase for Gaucher’s disease. N. Engl. J. Med..

[201] Vigan M., Stirnemann J., Caillaud C., Froissart R., Boutten A., Fantin B., Belmatoug N., Mentre F. (2014). Modeling changes in biomarkers in Gaucher disease patients receiving enzyme replacement therapy using a pathophysiological model. Orphanet J. Rare Dis..

[202] Weinreb N.J., Goldblatt J., Villalobos J., Charrow J., Cole J.A., Kerstenetzky M., vom Dahl S., Hollak C. (2013). Long-term clinical outcomes in type 1 Gaucher disease following 10 years of imiglucerase treatment. J. Inherit. Metab. Dis..

